# Two‐Photon Excited Near‐Infrared Phosphorescence Based on Secondary Supramolecular Confinement

**DOI:** 10.1002/advs.202201182

**Published:** 2022-04-24

**Authors:** Xin‐Kun Ma, Xiaolu Zhou, Jing Wu, Fang‐Fang Shen, Yu Liu

**Affiliations:** ^1^ College of Chemistry State Key Laboratory of Elemento Organic Chemistry Nankai University Tianjin 300071 P. R. China; ^2^ China Medical and Health Analysis Center Peking University Beijing 100191 P. R. China

**Keywords:** cucurbituril, NIR, phosphorescence, supramolecular assembly, two‐photon absorption

## Abstract

Organic phosphorescence materials have received wide attention in bioimaging for bio‐low toxicity and large Stokes. Herein, a design strategy to achieve near‐infrared (NIR) excitation and emission of organic room‐temperature phosphorescence through two‐stage confinement supramolecular assembly is presented. Via supramolecular macrocyclic confinement, the host–guest complexes exhibit phosphorescence with two‐photon absorption (excitation wavelength up to 890 nm) and NIR emission (emission wavelength up to 800 nm) in aqueous solution, and further nano‐confinement assembly significantly strengthens phosphorescence. Moreover, the nano‐assemblies possess color‐tunable luminescence spanning from the visible to NIR regions under different excitation wavelengths. Intriguingly, the prepared water‐soluble assemblies maintain two‐photon absorption and multicolor luminescence in cells or vivo.

## Graphical abstract

A multi‐stage assembly strategy involving macrocyclic confinement and nano‐confinement is reported, which induces and enhances guests’ phosphorescence with two‐photon absorption and NIR emission. Moreover, the assemblies possess excitation‐dependent emission properties, in which luminescence covers the visible and NIR regions under different excitation wavelengths.

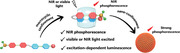

## 1 Introduction

Purely organic room‐temperature phosphorescence (RTP) materials have received increasing focus due to their extensive applications in the field of data recording and information security,^[^ ^1^ ^]^ organic light‐emitting diodes,^[^ ^2^ ^]^ and bioimaging.^[^ ^3^ ^]^ To improve luminescence performance, many brilliant organic RTP strategies have been developed, such as polymerization,^[^ ^4^ ^]^ crystallization,^[^ ^5^ ^]^ and supramolecular assembly.^[^ ^6^ ^]^ Among them, supramolecular assembly involving host–guest interactions and multi‐stages assembling is a convenient approach for improving phosphorescent performance and biocompatibility.^[^ ^7^ ^]^ Although there are numerous outstanding works, purely organic RTP materials in aqueous solutions, especially those that exhibit NIR emission, is still rare because the interference of oxygen and water inevitably lead to serious performance loss. Designing a large conjugate system with a donor–acceptor (D–A) construction is a common solution for red‐shift emission, however, this causes synthesis difficulties and poor water solubility, limiting their progress in bioimaging. Ma and co‐workers reported brominated phenolsulfonphthaleine derivatives that emit solid‐state NIR RTP with 3.0% quantum yield after doping into the polyvinyl alcohol rigid matrix.^[^ ^8^ ^]^ Two‐photon absorption is a way to achieve NIR‐excited luminescence. Wu and co‐workers reported difluoroboron *β*‐diketonate compounds achieving RTP via two‐photon NIR light excitation.^[^ ^9^ ^]^ Zhang et al. reported tetraphenylpyrrole derivatives with two‐photon excited phosphorescence, and nanoparticles were used for imaging.^[^ ^10^ ^]^ Nevertheless, to the best of our knowledge, there is no report on the purely organic RTP system with both NIR emission and excitation in an aqueous environment, where it remains a formidable challenge at present.

**Scheme 1:**
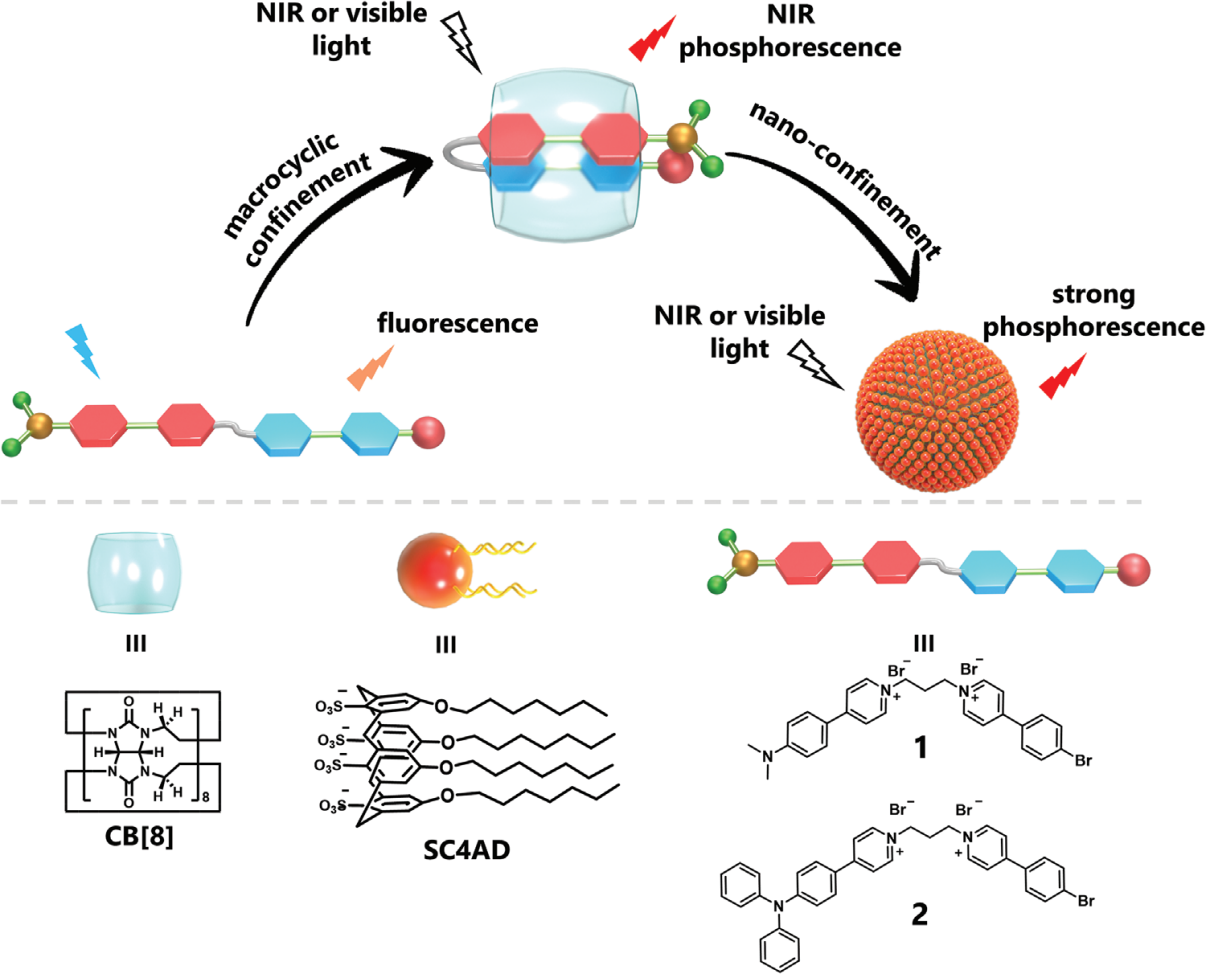
Schematic illustration of the formation of supramolecular assembly 1/CB[8]/SC4AD.

In this work, a series of phenylpyridinium salts with alkyl‐bridged D–A structure were synthesized (**Scheme** **1**). The strong binding of cucurbit[8]uril (CB[8]) promotes strong stacking between the donor moiety and acceptor moiety, which makes through‐space charge transfer (CT) possible. The nonconjugated CT promotes the phosphorescent emission redshift to the NIR region (*λ*_max_ up to 800 nm) and gives the property of two‐photon luminescence. Further assembly with amphiphilic macrocycle sulfonatocalix[4]arene (SC4AD) forms homogeneous spherical nanoparticles, resulting in a dramatic tenfold increase in phosphorescence emission intensity. Unexpectedly and more interestingly, the assemblies displayed excitation‐dependent luminescence, that is, at different excitation wavelengths, the assembly solution could exhibit tunable luminescence from green to yellow to red to NIR. Further studies revealed that supramolecular macrocyclic confinement is the key strategy for two‐photon NIR phosphorescence, and the second assembly not only enhances phosphorescence but promotes the formation of nanoparticles for cell imaging and in vivo mouse imaging with tissue penetration. This multi‐stage supramolecular confinement strategy provides a convenient approach to realizing NIR phosphorescence in an aqueous medium.

## 2 Results and Discussion

Alkyl‐bridged guests (1 and 2), which possess the same acceptor moiety (bromophenylpyridinium salt) and similar donor moiety (4‐(2‐pyridyl) aniline salt derivatives), were synthesized according to the routes shown in Scheme S1 (Supporting Information) and characterized by ^1^H NMR spectroscopy, ^13^C NMR spectroscopy, and high‐resolution mass spectrometry (HRMS) (Figures S1 and S2, Supporting Information).

**Figure 1:**
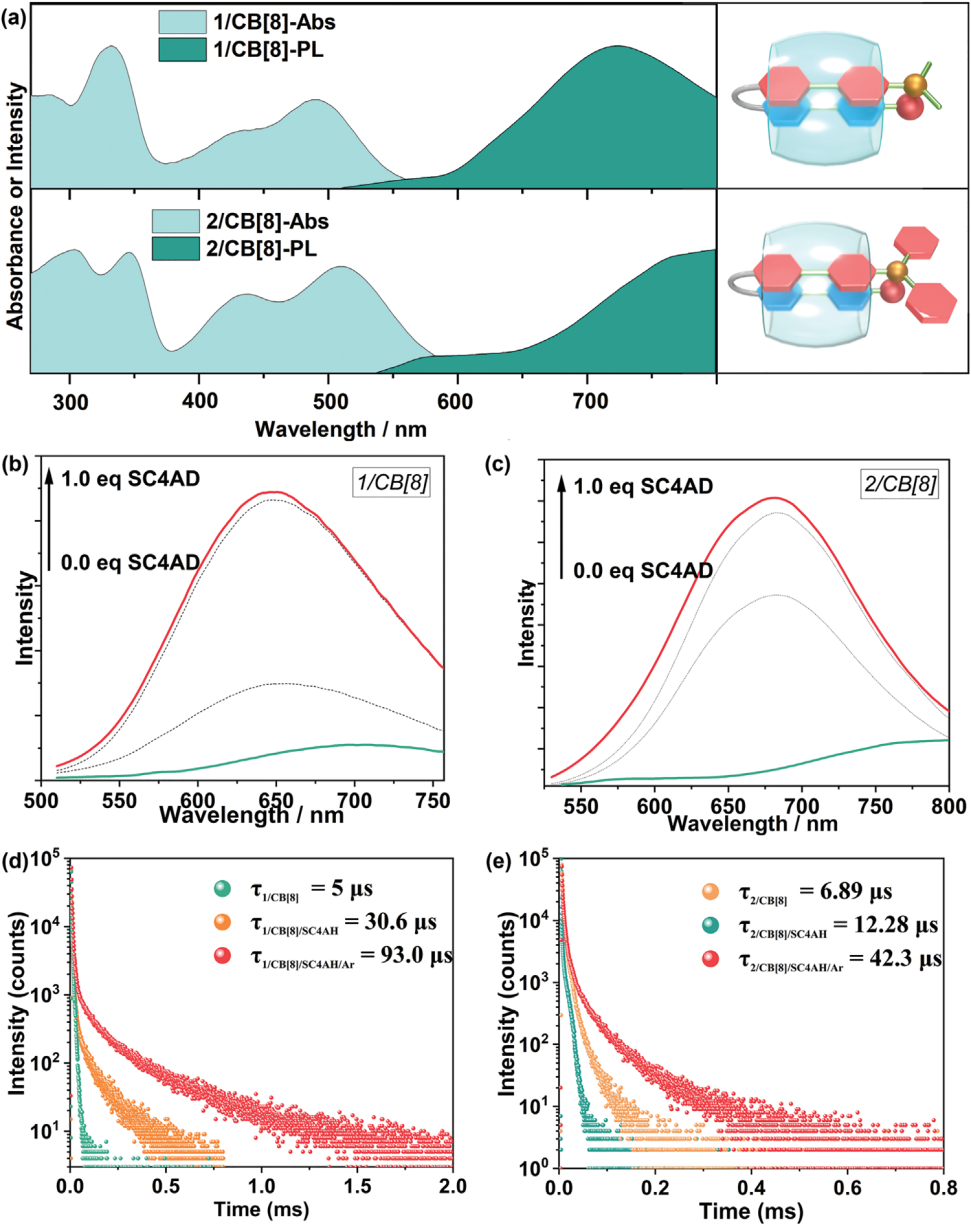
a) UV–vis absorption spectra and PL spectra of 1/CB[8] (top) and 2/CB[8] (button) complexes in aqueous solution, ([1] = [2] = [CB[8]] = 2 × 10^–5^ m, *λ*_ex_ of 1/CB[8] = 495 nm, *λ*_ex_ of 2/CB[8] = 510 nm); b,c) PL spectra of G/CB[8] (1/CB[8] or 2/CB[8]) upon the addition of 0, 0.5 × 10^–5^, 1.0 × 10^–5^, 1.2 × 10^–5^ m SC4AD in aqueous solution. (*λ*_ex_ of 1/CB[8] = 495 nm, *λ*_ex_ of 2/CB[8] = 510 nm, [1] = [CB[8]] = 2 × 10^–5^ m); d,e) Time‐resolved PL decay curves of G/CB[8], G/CB[8]/SC4AD, and G/CB[8]/SC4AD under argon in aqueous solution at 298 K ([G/CB[8]] = 2 × 10^–5^ m, [G/CB[8]/SC4AD] = 2 × 10^–5^ m, *λ*_em_ of 1/CB[8] = 725 nm, *λ*_em_ of 1/CB[8]/SC4AD = 650 nm, *λ*_em_ of 2/CB[8] = 800 nm, *λ*_em_ of 2/CB[8]/SC4AD = 675 nm).

The UV–vis absorption spectra of both unbound guests show similar peaks at ≈310 and 440 nm, which are assigned to the bromo‐substituted phenylpyridinium moiety and the *N*,*N*‐dimethyl‐substituted phenylpyridinium (Figure S4, Supporting Information). The steady‐state spectra of unbound guests exhibit yellow–green peaks named the yellow–green band. Further nanosecond‐scale lifetimes indicate that both emissions belong to fluorescence (Figures S5 and S6, Supporting Information). Meanwhile, no signs of phosphorescence were detected either in the delayed spectrum or in the time‐resolved photoluminescence decay (Figure S7, Supporting Information). After initially measuring the luminescent behavior of the unbound guest, the water‐soluble macrocycle compound CB[8], which has strong binding with a positively charged molecule and can accommodate two guest molecules, was added to the guests' aqueous solution. Interestingly, three clear isosbestic points were observed at ≈340, ≈350, and ≈480 nm in the UV−vis absorption spectrum along with new CT absorbance appearing at ≈500 nm (Figure S8, Supporting Information). In addition, the color of the solutions turns from pale yellow to red upon adding CB[8], which implies that new CT complexes were formed (Figure S8, Supporting Information). The dramatic variation in absorption drove us to investigate the luminescent properties. A new NIR band (550–800 nm, *λ*_1_ ≈ 725 nm, *λ*_2_ ≈ 800 nm) was observed in the PL spectra upon adding CB[8] (**Figure** **1**a). Immediately thereafter, a microsecond‐scale lifetime was detected at the maximum wavelength of the new emission peak, which implied the possibility of phosphorescence (Table S1, Figure 1d,e).

To achieve stronger luminescence in aqueous solutions, the host–guest complexes were co‐assembled with SC4AD, because SC4AD composed of hydrophobic alkyl chains and negatively charged sulfonate groups could payload positively charged guest molecules via electrostatic interactions. As expected, both phosphorescence intensities were significantly enhanced upon adding 1.0 equiv SC4AD, and the lifetime of phosphorescence was also enlarged several times (Figure 1b–e). For comparison, titration spectra of the guest alone with SC4AD were performed and no phosphorescent signal was detected (Figure S10, Supporting Information). These results indicated that supramolecular multi‐stage confinement assembly is an appropriate strategy for inducing and enhancing phosphorescence. We obtained the following additional evidence confirming that the NIR band emission is phosphorescence. First, the lifetimes of NIR emissions were increased markedly upon deoxygenation by bubbling argon, which is consistent with the fact that oxygen quenches triplet‐state electrons (Figure 1e). Then, low‐temperature PL and time‐resolved photoluminescence decay spectra further confirmed the previous assumption that the NIR band emission was greatly enhanced when the temperature decreased with increasing phosphorescence lifetime (Figure S11, Supporting Information). Third, the time‐resolved (delayed by 0.1 ms) photoluminescence spectra consistent with the PL spectra provided additional evidence for phosphorescent emission (Figure S12, Supporting Information). The above experiments confirm the RTP emission characteristics and dismiss the possibility of thermally activated delayed fluorescence.

**Figure 2:**
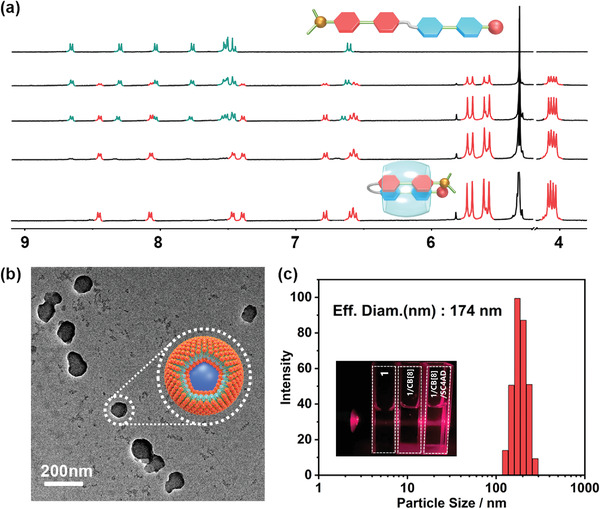
a) ^1^H NMR spectral changes of 1 after adding 0, 0.4, 0.6, 0.8, and 1.0 equivalent CB[8] (400 MHz, D_2_O, 298 K); b) TEM image of 1/CB[8]/SC4AD (G/CB[8]/SC4AD] = 2 × 10^–5^ m); c) DLS data 1/CB[8]/SC4AD ([1/CB[8]/SC4AD] = 2 × 10^–5^ m), insert: the Tyndall effect of 1(left), 1/CB[8](middle), and 1/CB[8]/SC4AD(right) ([1] = [1/CB[8]] = [1/CB[8]/SC4AD] = 2 × 10^–5^ m).

**Figure 3:**
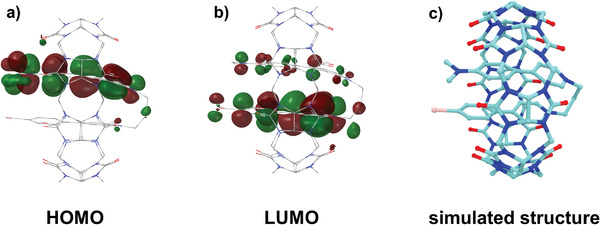
a) HOMO; b) LUMO; c) Simulated structure of the 1/CB[8].

To understand the mechanism for the phosphorescence generation and enhancement process, the assembly behaviors of CB[8] and SC4AD were determined. **1** as a typical compound was used to characterize the assembly behavior. UV–vis titration experiments were performed, and Job's plot was constructed based on the resulting data (Figure S13, Supporting Information). The plot indicated that the 1 and CB[8] binding stoichiometry was 1:1, and the association constant (*K*_s_) was determined to be 3.88 × 10^7^ m ^−1^ in an aqueous solution (Figure S14, Supporting Information). Further evidence for the formation of a 1:1 host–guest assembly came from high‐resolution ESI‐TOF MS (Figure S15, Supporting Information), and the intensity peak for host/guest complexes matched the calculated 1:1 value well. Same conclusion was also confirmed by means of two‐dimensional NMR analysis. Specifically, NOESY (nuclear overhauser effect spectroscopy) of **1**/CB[8] showed strong rotating frame nuclear Overhauser effect signals between the pyridinium groups (Figure S18a, Supporting Information), which indicated that the form of the assemblies was “head‐to‐head binding.” ^1^H NMR spectroscopy provided more details about binding behaviors: chemical shifts of pyridine salts and phenyl protons of **1** shifted to the higher field with the alkyl protons’ chemical shifts moved to the lower field, which indicated that the aromatic rings were enclosed in the CB[8] cavity, whereas the flexible alkyl chain was outside the cavity (**Figure** **2**a). Density functional theory (DFT) calculations provide visualized images of the host–guest complex. In **Figure** **3**c, the simulated structure shows that the bromo‐substituted moiety folds back and stacks with the *N*,*N*‐dimethyl‐substituted‐substituted phenylpyridinium moiety. This formation might result from hydrophobic effects, *π*−*π* stacking, and CT interactions. Combined with the results of UV–vis spectra titrations, high‐resolution MS, ^1^H NMR spectroscopy, and DFT calculations, we confirmed that 1 folded into the cavity of CB[8] to form a 1:1 single molecular heterodimer assembly. 2 was performed with the same experiments and showed the same 1:1 and molecular fold results. The progress of second‐stage assembly with SC4AD was demonstrated by Tyndall experiments, transmission electron microscopy (TEM), dynamic light scattering (DLS) and zeta potential experiments. TEM measurements showed that 1/CB[8]/SC4AD was a similarly sized spherical nanoparticle with a diameter of ≈100 nm, and the DLS experiment provided similar particle size results (≈174 nm) (Figure 2b,c). The average zeta potential of 1/CB[8] was +1.96 mV, and that of 1/CB[8]/SC4AD was −17.75 mV (Figure S21a,b, Supporting Information), indicating the formation of a stable assembly after the addition of SC4AD. These results suggested that the host–guest complexes form stable nanoaggregates with SC4AD through electrostatic and hydrophobic interactions, which is consistent with a previously reported system.^[^ ^11^ ^]^

What is the role of the macrocycles in the generation and conversion of triplet electron? It has been widely reported that macrocyclic compounds such as cyclodextrin and cucurbituril are able to capture the guests firmly and simultaneously suppress nonradiative relaxation processes, thus endowing supramolecular luminescent assemblies with high RTP efficiencies.^[^ ^12^ ^]^ Apart from limiting the motion of molecules, in our system, the binding of CB[8] promotes intramolecular charge transfer via *π*–*π* stacking. In UV–vis absorption spectra, a new CT absorbance at ≈500 nm was observed upon adding CB[8] (Figure S8, Supporting Information); in contrast, guest 3 without the D–A structure only displayed a redshift after binding with CB[8] (Figure S22, Supporting Information). To exclude the possibility of a bathochromic shift from J‐aggregation, experiments on solvent polarity‐dependent PL emission were constructed (Figure S23, Supporting Information). As the solvent polarity decreased, the NIR band emission had a significant blueshift, which further proved that CT assembly formed upon binding with CB[8]. Solvent polarity‐dependent PL emission provided a reasonable and possible explanation for the host–guest complex being loaded in the SC4AD hydrophobic environment. The process of intramolecular CT was reproduced by TD‐DFT calculations and can be explained by the frontier molecular orbitals in 1 and 1/CB[8]. As shown in the Figure 3 and Figure S33 (Supporting Information), the HOMO and LUMO of 1/CB[8] and 2/CB[8] are almost entirely separated. The occupied and virtual molecular orbitals are more localized at the *N*,*N*‐dimethylpyridine, respectively, unoccupied molecular orbitals are more localized at the Br‐dimethylpyridine, rendering a possible *N*,*N*‐dimethylpyridine‐to‐Br‐dimethylpyridine charge transfer character. Through‐space charge transfer will contribute to reducing singlet–triplet energy splitting, which further promotes phosphorescence by accelerating ISC.^[^ ^13^ ^]^

**Figure 4:**
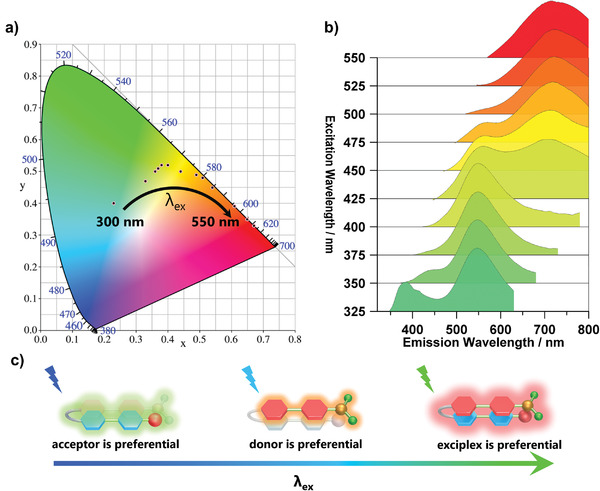
a) CIE 1931 chromaticity diagram of assemblies 1/CB[8] in aqueous solution with different excitation wavelengths under ambition conditions (from 300 to 550 nm, [1/CB[8]] = 2 × 10^–5^ m); b) PL spectra of 1/CB[8] ([1/CB[8]] = 2 × 10^–5^ m) in aqueous solution under ambient conditions excited with different wavelengths (from 325 to 550 nm); c) Possible mechanism of excitation‐dependent luminescence.

Macrocycle‐enhanced through‐space CT not only energizes phosphorescence but also results in excitation‐dependent emission characteristics. When the excitation wavelength is redshifted from 300 to 550 nm, the 1/CB[8] solution luminescence color transforms from green to orange and finally to red. **Figure** **4** shows that the relative intensity of the blue, green, and red bands determines the luminescence color. Compared with reference compounds 1–2 and 1–3, the blue band was assigned to the Br‐dimethylpyridine salt moiety, the yellow band was assigned to the *N*,*N*‐dimethylpyridine salt moiety, and the NIR band was assigned to the CT complex (Figures S24 and S25, Supporting Information). To avoid the possibility that excitation‐dependent emission results from multiple luminescence centers caused by the guest not being fully encapsulated, NMR titration experiments were performed (Figure 2a). The signal of unbound guest disappears completely when the concentration of CB[8] is 1.0 equivalent. In addition, the fluorescence lifetime provides another evidence that excitation‐dependent luminescence originates from the host–guest complex. In both the green and blue bands, the lifetimes of the host–guest complexes are longer than those of the guests (Figure S26, Supporting Information). After confirming the luminescent center, a possible mechanism was presented, and the donor moiety, acceptor moiety, and CT exciplex moiety could be excited separately. When the excitation wavelength was below 350 nm, the bromo‐substituted phenylpyridinium moiety and *N*,*N*‐dimethylpyridine salt moiety were separately and preferentially activated; hence, the G/CB[8] solution mainly showed green emission. After 350 nm, the *N*,*N*‐dimethylpyridine salt moiety was preferentially excited, and the intensity of the yellow emission became predominant. When the excitation wavelength was above 500 nm, the CT complexes were energized, which led to a shift from orange to red emission (Figure 4 and Figure S27, Supporting Information). Moreover, the secondary assemblies G/CB[8]/SC4AD exhibit similar excitation‐dependent luminescence properties (Figures S28 and S29, Supporting Information). Compared with G/CB[8], the excitation‐dependent luminescence properties of G/CB[8]/SC4AD are inconspicuous, This may be due to the fact that the nano‐confinement from amphiphilic macrocycle greatly promotes phosphorescence; as a comparison, the intensity of fluorescence does not change significantly (Figure S30, Supporting Information).

**Figure 5:**
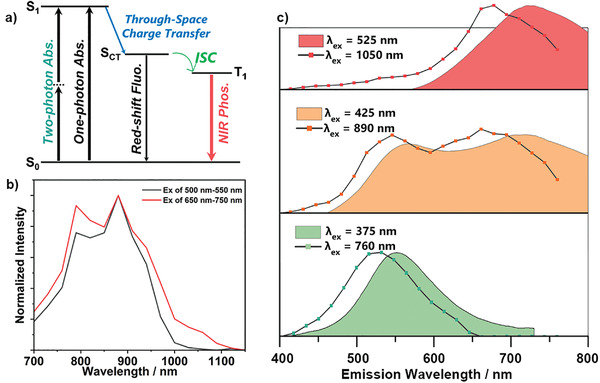
a) A possible diagram of the mechanism of NIR excitation and emission for the G/CB[8]/SC4AD systems (abs. = absorption, fluo. = fluorescence, non. ISC = intersystem crossing, phos. = phosphorescence). b) Two‐photon excitation spectrum of 1/CB[8] in aqueous solution under ambition conditions ([1/CB[8]] = 2 × 10^–5^ m). c) PL spectra of assembly 1/CB[8] excited with different wavelengths in aqueous solution under ambition conditions ([1/CB[8]] = 2 × 10^–5^ m). (Dotted line diagram is two‐photon emission spectra, and folded line chart is one‐photon emission spectra).

It is well‐known that large conjugation length systems with a strong intramolecular charge transfer effect are conducive to two‐photon absorption properties.^[^ ^14^ ^]^ This drives us to find out whether macrocycle‐induced space‐through CT possesses the two‐photon luminescence property. Encouragingly, the excitation wavelength range for 1/CB[8] is 700–1000 nm when using a 500–550 nm filter, while the excitation range is 700–1100 nm when using a 650–750 nm filter (**Figure** **5**b). It must be mentioned that a new excitation band (NIR‐II band) appears near 1050 nm when using NIR filter, and PL spectra with different excitation wavelengths reveal the source of the near 1050 nm excitation band. Green band emission was found when the 1/CB[8] solution was excited at 760 nm. Then, the excitation wavelength was redshifted to 890 nm, and the solution was found to emit a green band and NIR emission. When the excitation was redshifted to 1050 nm, the NIR band luminescence was preferentially obtained (Figure 5c). This implies that the NIR‐II excitation band originates from the two‐photon absorption of the CT absorption near 500 nm. Therefore, the assemblies have both two‐photon luminescence and tunable luminescence under the excitation of NIR lasers. In addition, the secondary assembly also possesses tunable luminescence for near‐infrared excitation (Figure S31, Supporting Information).

**Figure 6:**
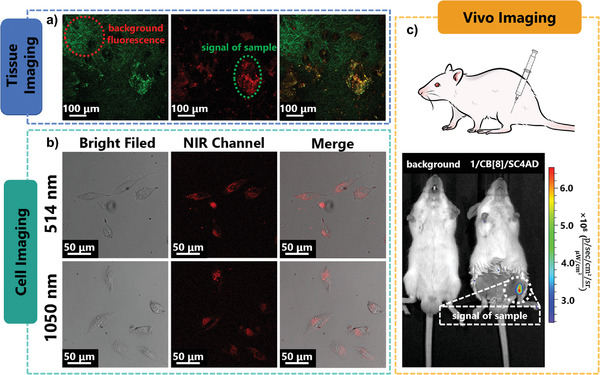
a) Vivo mouse tissue imaging of background fluorescence interference (left), 1/CB[8]/SC4AD (middle), and merged image (right). (The excitation wavelength is 1050 nm. The emission wavelength ranges for the green and NIR channels were 450–530 nm and 650–750 nm, respectively). b) Cell imaging of living A549 cells incubated with 1/CB[8]/SC4AD using a 514 nm excitation light and a 1050 nm femtosecond pulsed laser. (The emission wavelength ranges for NIR channels were 650–800 nm). c) In vivo living mouse imaging of 1/CB[8]/SC4AD ([1/CB[8]] = 1 × 10^–4^ m, *λ*_ex_ = 465 nm, *λ*_em_ = 630–670 nm).

Owning to its excellent NIR‐excited NIR phosphorescence, 1/CB[8]/SC4AD supramolecular assembly is a satisfactory candidate for bioimaging. When 1/CB[8]/SC4AD was incubated with A549 cells for 24 h, the 80% survival rate indicated that the assemblies had low cytotoxicity (Figure S34, Supporting Information). For confocal laser scanning microscopy (CLSM) imaging, the cells were stained with 1/CB[8]/SC4AD (10 × 10^−6^ m). In **Figure** **6**b, bioimaging of cells incubated with 1/CB[8] by CLSM suggested that assemblies could be efficiently internalized into the cell and displayed effective red phosphorescence. In addition, the assembly maintains excitation‐dependent properties in the cell environment (Figure S35, Supporting Information). Under a 405 nm excitation light, the green and blue channels displayed bright signals, while a shimmering signal was detected in the NIR channel. When the excitation wavelength is redshifted to 488 nm and then to 514 nm, the NIR signal becomes more intense, and the green channel signal becomes weaker. That is, these assemblies could achieve cell imaging with different channels of luminescence by changing the excitation wavelength. Compared with one‐photon imaging, two‐photon luminescence imaging excited by a NIR femtosecond pulsed laser displays greater performance with a higher signal‐to‐noise ratio. Photoluminescence photographs of 1/CB[8]/SC4AD from two‐photon imaging irradiated with a 1050 nm femtosecond pulsed laser exhibit excellent overlap compared to one‐photon imaging with a 514 nm laser (Figure 6b). Owing to NIR emission and excitation, 1/CB[8]/SC4AD is a potential candidate for imaging at the cellular level as well as in tissues and living bodies. 1/CB[8]/SC4AD (1 × 10^–4^ m ^–1^, 100 µL) was subcutaneously injected into live mice for investigating in vivo imaging. Under excitation by a femtosecond pulsed laser (1050 nm), the NIR phosphorescence signal can be clearly observed through the skin in Figure 6a; moreover, NIR luminescence can prevent the self‐fluorescence interference of biological tissues. In vivo imaging was performed using an IVIS system. The assemblies 1/CB[8]/SC4AD (1 × 10^–4^ m ^–1^, 100 µL) were injected into the leg of the mouse, and the NIR signal was still visible after 1 h, which indicated that the assemblies remained stable within vivo.

## 3 Conclusion

We constructed a multi‐stage assembly supramolecular system with NIR excitation and NIR phosphorescence emission. The through‐space charge transfer enhanced by the macrocycle not only promotes NIR phosphorescence but also facilitates excitation‐dependent tunable emission. Preliminary two‐photon luminescence cell and in vivo imaging experiments illustrate the great potential of supramolecular strategies for the development of biological theragnostic.

## Conflict of Interest

The authors declare no conflict of interest.

## Supporting information

Supporting Information

## Data Availability

The data that support the findings of this study are available from the corresponding author upon reasonable request.
