# Supplementary material for: Two‐Photon Excited Near‐Infrared Phosphorescence Based on Secondary Supramolecular Confinement
Source: Adv Sci (Weinh). 2022 Apr 24;9(18):2201182. doi: 10.1002/advs.202201182 (PMC9218752; doi:10.1002/advs.202201182)
Supplement: Supplementary file 1 — Supporting Information [file ADVS-9-2201182-s001.pdf]

ADVANCED SCIENCE
Open Access

Supporting Information

for *Adv. Sci.*, DOI 10.1002/advs.202201182

Two-Photon Excited Near-Infrared Phosphorescence Based on Secondary Supramolecular Confinement

*Xin-Kun Ma, Xiaolu Zhou, Jing Wu, Fang-Fang Shen and Yu Liu**

Electronic Supplementary Information (ESI)

Two-Photon Excited Near-Infrared Phosphorescence Based on Secondary Supramolecular Confinement

CONTENT

Section A. Materials and Methods 2

Section B. Synthesis and Characterization 3

Section C. Characterization and Photophysical Properties of Assemblies 9

Section D. Theoretic Calculations 22

Section E. Cells Experiments 23

Section F. Reference 24

Section A. Materials and Methods

All reagents and solvents were purchased from commercialized suppliers and used as supplied except for specifying otherwise noted. ^{1}H NMR and ^{13}C NMR were performed with an Ascend 400 MHz instrument. NOESY and COSY spectra were measured on a ZhongKe-Oxford I-400 instrument. High-resolution mass spectrometry (HRMS) was recorded on a Q-TOF LC-MS in electrospray ionization mode. UV-vis absorption spectra were recorded on a Shimadzu UV-3600 spectrophotometer with a PTC-348WI temperature controller in a quartz cell (light path 10 mm) at 298 K. Photoluminescence (PL) spectra, time-correlated decay profiles, quantum efficiency were processed on an Edinburgh Instruments FLS980 (Livingstone, UK). TEM experiments were carried out on an FEI Tecnai G2 F20 microscope operating at 200 KV. Dynamic Light Scattering (DLS) was examined by using a laser lights-cattering spectrometer (BI-200SM) equipped with a digital correlator (Turbo Corr) at 636 nm at a scattering angle of 90°. The hydrodynamic diameter (Dh) was determined by DLS experiments at 298 K. Zeta potential analysis was performed on a Brookhaven ZetaPALS (Brookhaven Instrument, USA) at 298 K. The cell images were captured on Leika S8 microscope and Olympus FV1000 Laser scanning confocal microscope. All measurements were carried out at room temperature (RT) except for specifying otherwise. The Commission International del'Eclairage (CIE) 1931 chromaticity diagram was obtained on FLS980.

Theoretical calculations

All quantum-chemical calculations including density functional theory (DFT) and time-dependent density functional theory (TD-DFT) calculations were performed by Gaussian 09 program packages. The visualization and distribution of molecular orbital were analyzed by GaussView.

Cytotoxicity experiments and Cell imaging

The human lung adenocarcinoma cells (A549) were obtained from the Cell Resource Center of China Academy of Medical Science in Beijing. The culture conditions for A549 cells: Ham's F12 nutrient medium supplemented with 10 % FBS and 1 % penicillin/streptomycin and humidified incubator with 5% CO_2 atmosphere at 37 °C. A549 cells were seeded in 96-well plates for 12 h at 37 °C in 5% CO_2. The A549 cells were incubated with G/CB[8]/SC4AD at different concentrations in 96-well plates for 24 h. The relative cellular viability was determined by the CCK8 assay.

The A549 cells were seeded in a confocal petri dish and cultured in a humidified incubator at 37 °C with 5% CO_2 for 24 hours. The well-cultured cells were incubated with G/CB[8]/SC4AD (2×10^{-5} M) for 12 h. The cells were then repeatedly washed at least three times with PBS and observed with a confocal microscope. The two-photon imaging of G/CB[8]/SC4AD in cells was recorded with Leika S8 confocal microscope.

In vivo imaging

All experimental procedures with animals were carried out by licensed investigators. Animals: One-month-old male ICR mouse (20g), were obtained from the Department of Laboratory Animal Science, Peking University Health Science Center. Feeding conditions: All the animals were submitted to controlled temperature conditions (22~26 °C) humidity (50~60%), light (12 h light/12 h dark, 15~20 LX), adequate water, and adequate food. And all the animals were maintained on the standard laboratory-specific pathogen-free (SPF). In vivo imaging: The living mice were anesthetized with Isoflurane.

G/CB[8]/SC4AD (1×10^{-4} M) was injected subcutaneously into living mice. The live imaging data were captured on the IVIS Spectrum

Section B. Synthesis and Characterization

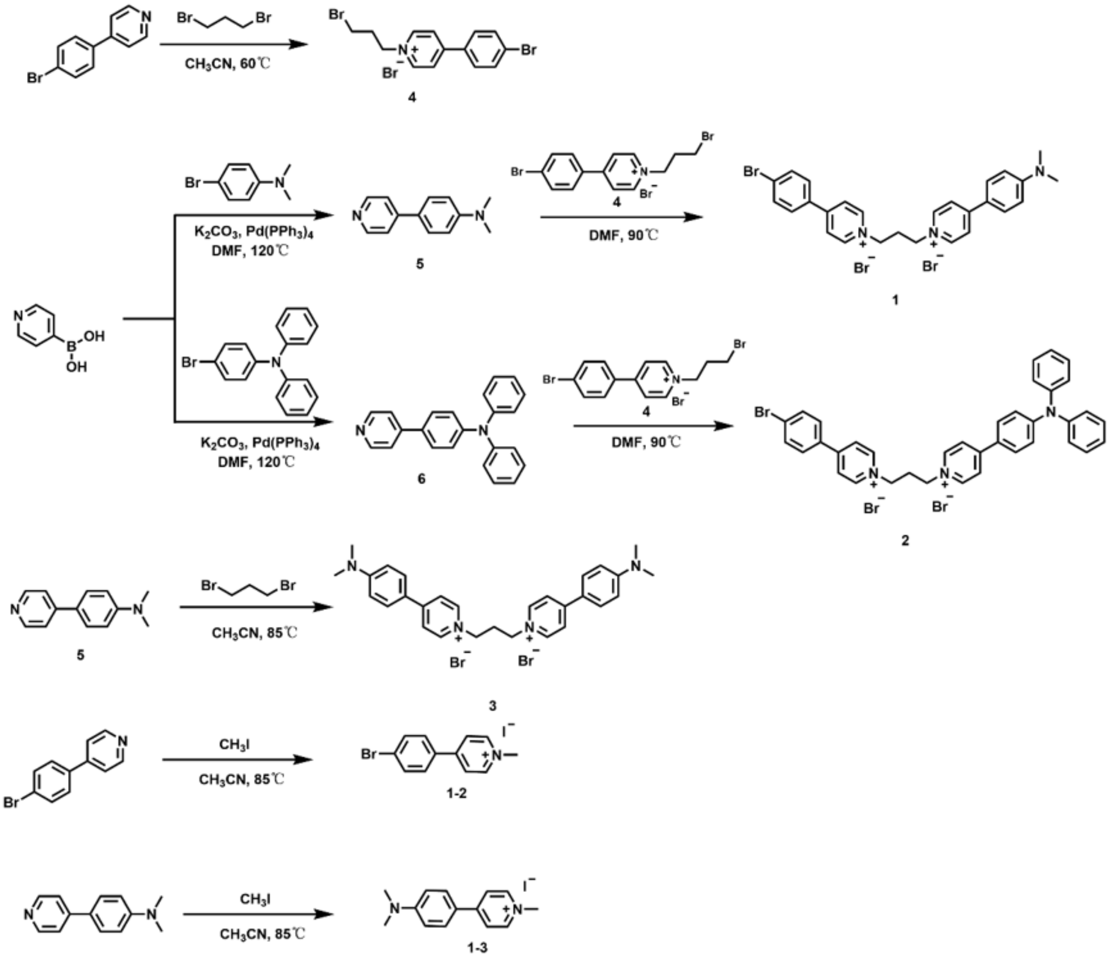

Scheme S1. Synthetic routes of **1**, **2, 3, 1-2 and 1-3**.

Synthesis of compound 4:

Compound 4 was synthesized according to the literature.[1] 4-(4-bromophenyl)pyridine (100 mg, 0.43 mmol) and 1,3-dibromopropane (0.35 g, 1.74 mmol) were dissolved in 25 mL CH_3CN and heated at 60 °C for 12 h. After being cooled to room temperature, the reaction mixture was dispersed in 250 mL diethyl ether. The mixture was filtered and the solid was washed with acetone. The product was obtained as white solid (120 mg, 65%).

Synthesis of compound 5:

Compound 5 was synthesized according to the literature.[2] 4-bromo-N,N-dimethylaniline (0.8 g, 4 mmol), 4-pyridineboronic acid (0.55 g, 4.4 mmol), K_2CO_3 (0.83 g, 6.0 mmol) were dissolved in anhydrous DMF (40 ml) and degassed for 30 min. Then Tetrakis(triphenylphosphine)palladium (0.19 g, 0.16 mmol) was rapidly added to the solution and degassed continued for an additional 15 min. The reaction mixture was heated at 110 °C under an argon atmosphere for 36 h. The crude was poured into water (50 mL) and extracted with CH_2Cl_2 (3×20 mL). The organic layer was washed with saturated aqueous sodium bicarbonate solution (3×15 mL) and saturated brine (3×20 mL), dried over anhydrous $MgSO_4$, filtered, and concentrated by rotary evaporation. The resulting residue was further purified by silica gel chromatography using petroleum ether: ethyl acetate (5:1, V/V) as eluent, compound 5 was obtained as white solid (0.47 g, 59 %).

Synthesis of compound 6:

Compound 6 was synthesized according to the literature[3] and is similar to the description of compound 5. Product was obtained as white solid (65%).

Synthesis of compound 1:

Compound 5 (50 mg, 0.25 mmol) and compound 4 (100 mg, 0.23 mmol) were dissolved in 10 mL DMF and heated at 90 °C for 48 h. The residue was cooled to room temperature and evaporated to remove the solvent. The crude product was recrystallized with CH_3CN to obtain the yellow powder with a yield of 42% (60 mg). ^{1}H NMR (400 MHz, D_2O) δ 8.64 (d, *J* = 6.9 Hz, 2H), 8.28 (d, *J* = 7.1 Hz, 2H), 8.01 (d, *J* = 6.9 Hz, 2H), 7.74 (d, *J* = 7.1 Hz, 2H), 7.50 (dd, *J* = 9.0, 2.9 Hz, 4H), 7.46 – 7.42 (m, 2H), 6.59 (d, *J* = 9.2 Hz, 2H), 4.78 (s, 2H), 4.66 – 4.61 (m, 2H), 3.00 (s, 6H), 2.85 – 2.80 (m, 2H).； ^{13}C NMR (101 MHz, DMSO-d_6) δ 154.08, 153.67, 153.05, 145.08, 143.68, 132.62, 130.12, 129.62, 126.25, 124.51, 120.94, 118.59, 112.15, 56.89, 55.69, 39.62, 31.41.; HRMS (ESI) m/z for $C_{27}H_{28}Br_3N_3$ calcd. $[M-2Br]^{2+}$ 236.5728, found: 236.5730.

Synthesis of compound 2:

Similar to the synthesis of compound 1, compound 2 was obtained as red-brown powder with 31% yield (54 mg) with compound 6 (80.5 mg, 0.25 mmol) and compound 4 (100 mg, 0.23 mmol) as raw material. ^{1}H NMR (400 MHz, DMSO-d_6) δ 9.13 (d, *J* = 6.5 Hz, 2H), 8.93 (d, *J* = 6.5 Hz, 2H), 8.59 (d, *J* = 6.4 Hz, 2H), 8.40 (d, *J* = 6.6 Hz, 2H), 8.05 (d, *J* = 8.7 Hz, 2H), 8.00 (d, *J* = 8.9 Hz, 2H), 7.92 – 7.87 (m, 2H), 7.48 – 7.42 (m, 4H), 7.28 – 7.19 (m, 6H), 6.97 (d, *J* = 8.9 Hz, 2H), 4.71 (t, *J* = 7.2 Hz, 2H), 4.64 (t, *J* = 7.2 Hz, 2H), 2.70 (s, 2H).; ^{13}C NMR (101 MHz, DMSO-d_6) δ 154.33, 154.18, 151.71, 146.04, 145.62,

144.85, 133.22, 133.10, 130.55, 130.17, 126.83, 126.63, 125.85, 124.99, 124.64, 122.98, 119.84, 57.35, 56.69, 31.90.; HRMS (ESI) m/z for $C_{37}H_{32}Br_3N_3$ calcd. $[M-2Br]^{2+}$ 298.5885, found: 298.5887.

Synthesis of compound 3:

Compound 5 (100 mg, 0.5 mmol) and 1,3-dibromopropane (40 mg, 0.2 mmol) were dissolved in 10 mL CH_3CN and heated at 85 °C for 24 h. An orange precipitate separates out from the reaction solution and then filtered, washed with acetone (3×10 ml), and dried in vacuo. Pure product was obtained as orange powder (76.3 mg, 81%). ^1H NMR (400 MHz, D_2O) δ 8.23 (d, J = 6.7 Hz, 4H), 7.73 (d, J = 6.7 Hz, 4H), 7.47 (d, J = 8.9 Hz, 4H), 6.53 (d, J = 8.8 Hz, 4H), 4.60 (t, J = 5.7 Hz, 4H), 2.94 (s, 12H), 2.76 (d, J = 6.1 Hz, 2H).; ^{13}C NMR (101 MHz, DMSO-d_6) δ 154.63, 153.59, 144.20, 130.14, 127.83, 121.45, 112.69, 56.36, 40.14, 31.78.; HRMS (ESI) m/z for $C_{29}H_{34}Br_2N_4$ calcd. $[M-2Br]^{2+}$ 219.1386, found: 219.1389.

Synthesis of compound 1-2, 1-3:

Compound 1-2 was prepared according to the previous work.[4] Compound 1-3 was prepared according

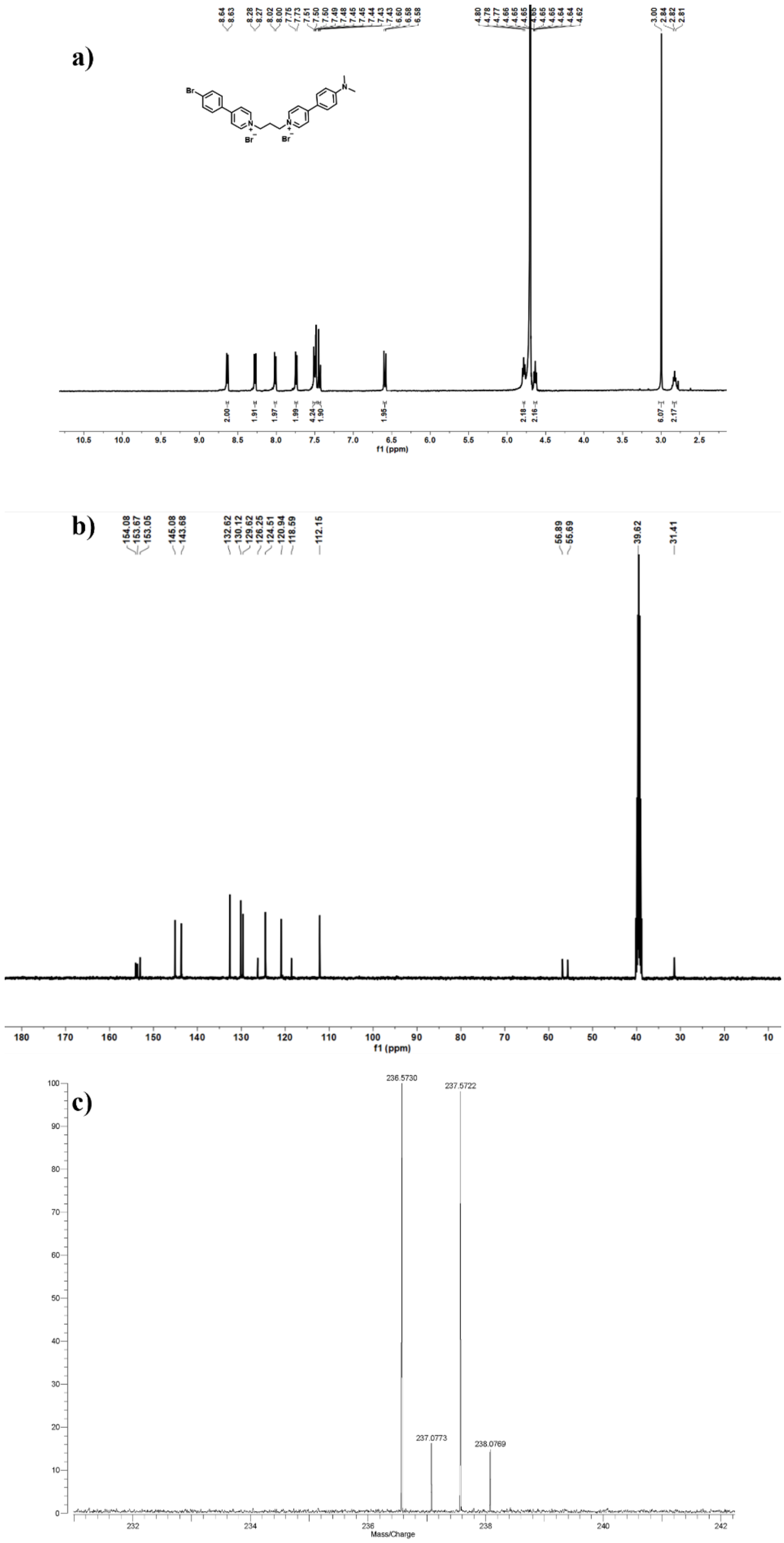

Figure S1. (a) ^{1}H NMR spectrum (400 MHz, D_2O, 298 K), (b) ^{13}C NMR spectrum (400 MHz, DMSO-*d6*, 298 K), (c) HRMS (ESI) spectrum of compound 1.

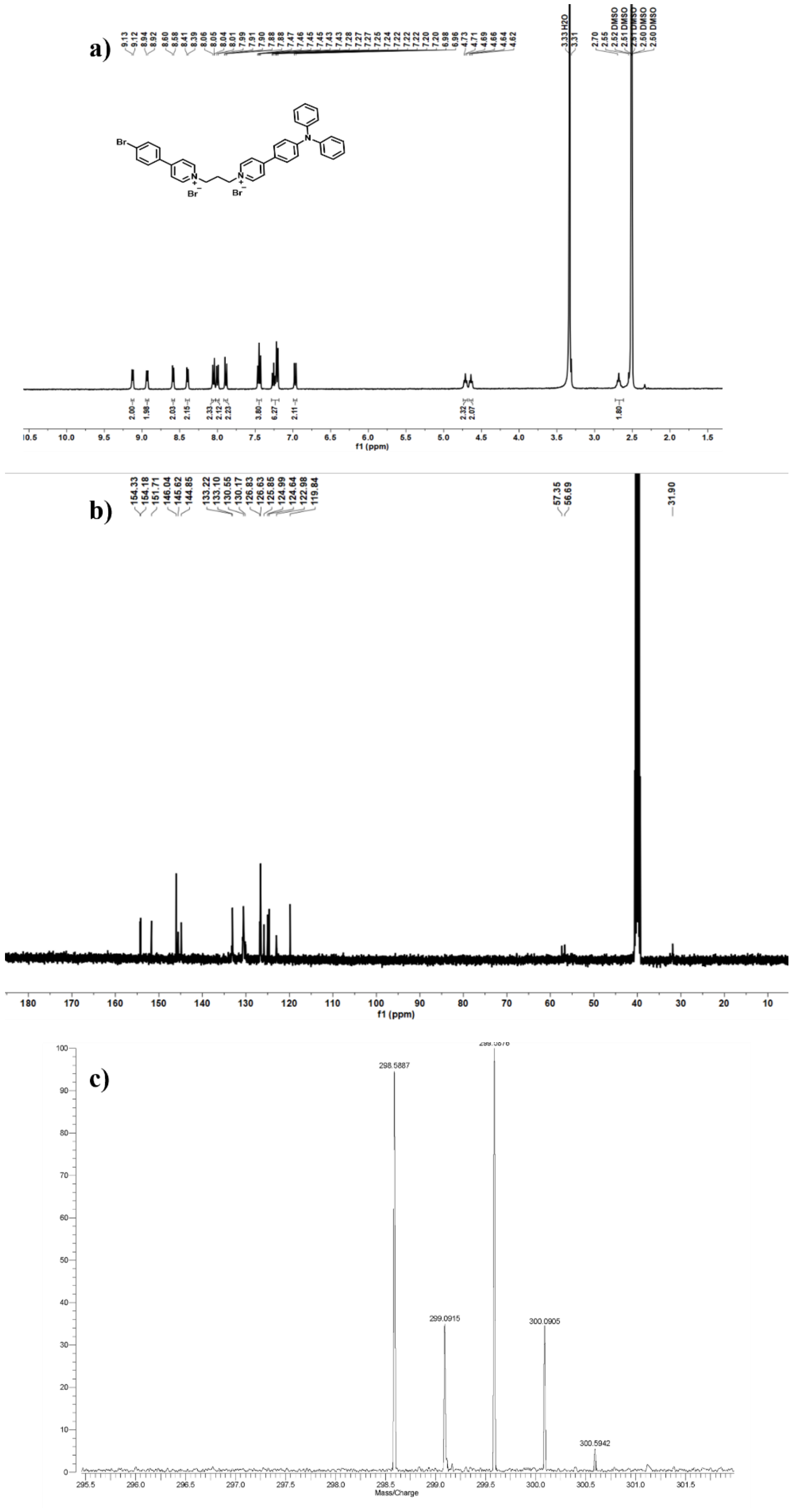

Figure S2. (a) ^{1}H NMR spectrum (400 MHz, DMSO-*d6*, 298 K), (b) ^{13}C NMR spectrum (400 MHz, DMSO-*d6*, 298 K), (c) HRMS (ESI) spectrum of compound 2.

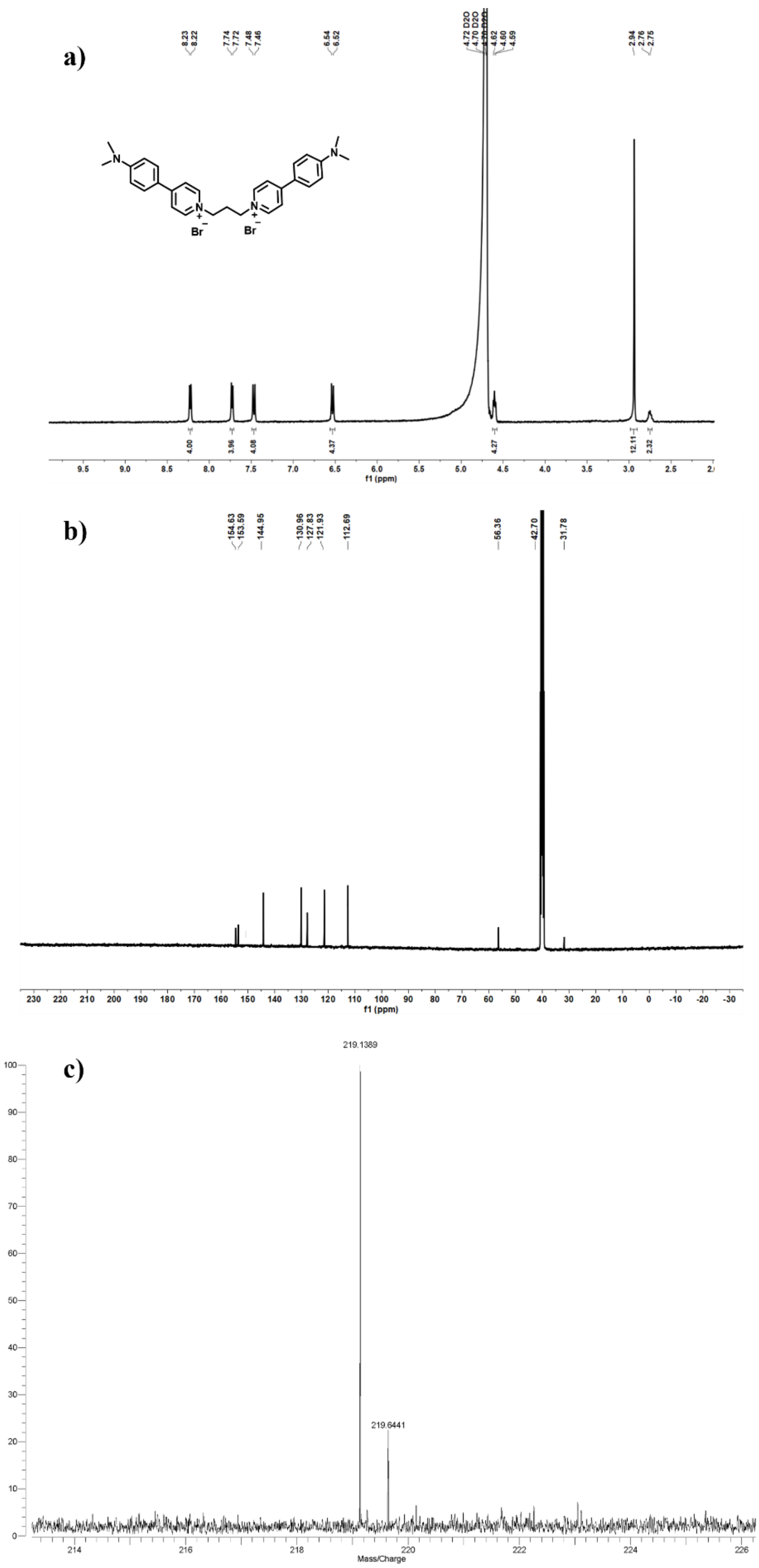

Figure S3. (a) ^{1}H NMR spectrum (400 MHz, D_2O, 298 K), (b) ^{13}C NMR spectrum (400 MHz, DMSO-*d6*, 298 K), (c) HRMS (ESI) spectrum of compound 3.

Section C. Characterization and Photophysical Properties of Assemblies

Table S1 Photophysical data for compound in aqueous solution

compound	λ_{ex} (nm)	$\lambda_{flu.}$ (nm)	$\lambda_{pho.}$ (nm)	$\tau_{Flu.}$ (ns)	$\tau_{Pho.}$ (μs)	Φ (%)
**1**	305/440	390/530	ND	0.94(390nm)<br>1.72(530nm)	ND	ND
**1/CB[8]**	495	390/530	720	1.93(390nm)<br>4.88(530nm)	5.00	0.35
**1/CB[8]/SC4AD**	495	ND	670	ND	30.6	5.08
**2**	300/440	390/620	ND	1.05(390nm)<br>4.10(620nm)	ND	ND
**2/CB[8]**	510	390/620	800	1.80(390nm)<br>6.20(620nm)	6.89	0.70
**2/CB[8]/SC4AD**	510	ND	680	ND	12.28	9.30
**3**	440	530	ND	1.28	ND	ND

ND: not detected

λ_{ex} : Excitation wavelength

$\lambda_{flu.}$: Fluorescence emission wavelength

$\lambda_{pho.}$: Phosphorescence emission wavelength

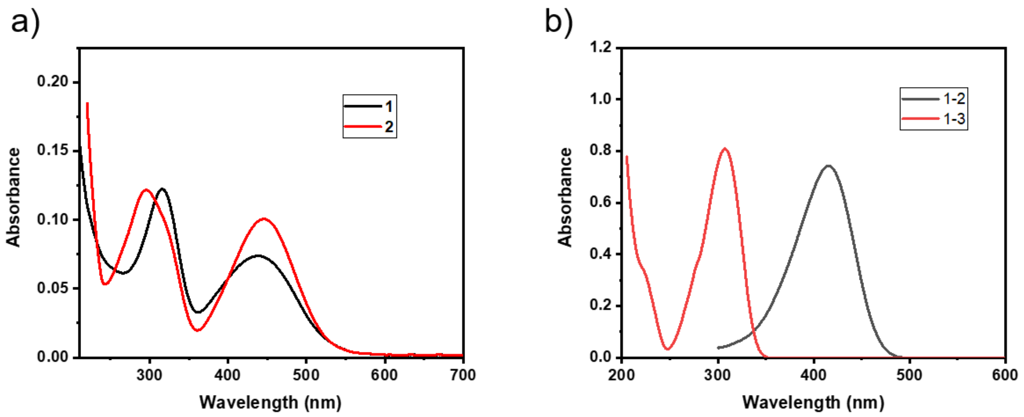

Figure S4. (a) The UV–vis absorption spectrum of compound 1 and 2 in aqueous solution at 298 K ([1]=[2]=10 μM); (b) The UV–vis absorption spectrum of compound 1-2 and 1-3 in aqueous solution at 298 K ([1-2] = [1-3] = 10 μM).

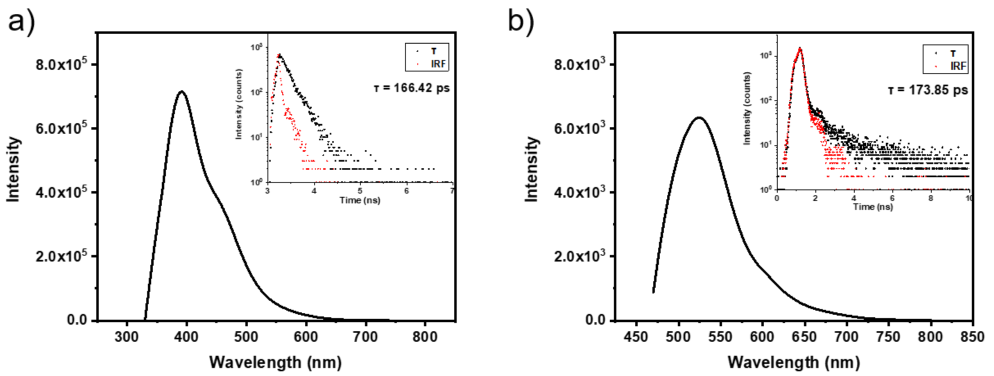

Figure S5. The steady-state spectra of unbound guest 1 under ambition condition (a: λ_{ex} = 305 nm; b: λ_{ex} = 440 nm, [1] = 10 μM).

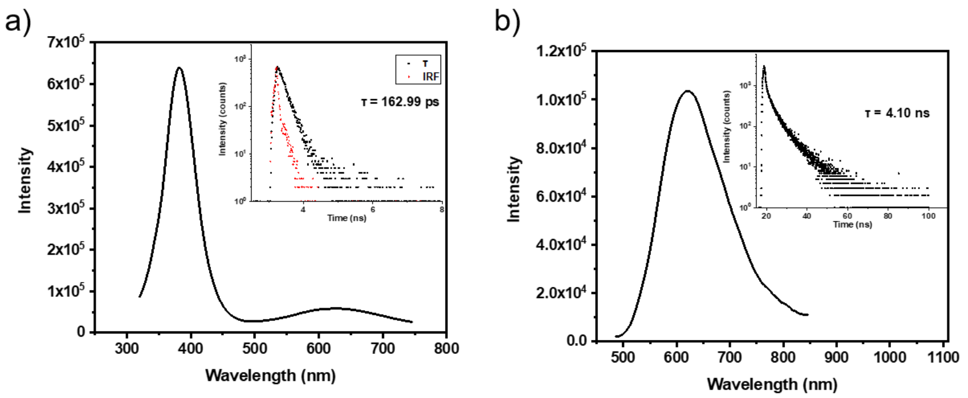

Figure S6. The steady-state spectra and time-correlated decay curves of unbound guest 2 under ambition condition (a: λ_{ex}= 300 nm; b: λ_{ex}= 450 nm, [2] = 10 μM).

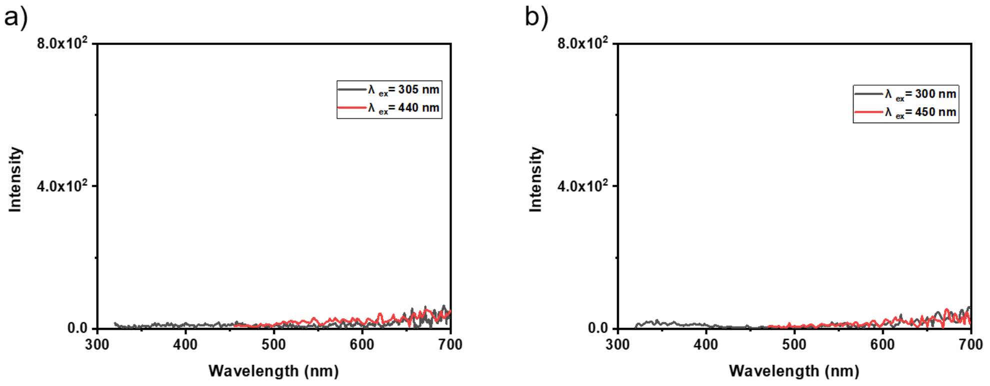

Figure S7. (a) The delayed spectrum of guest 1 (black line: λ_{ex}= 305 nm; red line: λ_{ex}= 440 nm, delayed time = 50 μs, [1] = 10 μM). (b) The delayed spectrum of guest 2 (black line: λ_{ex}= 300 nm; red line: λ_{ex}= 450 nm, delayed time = 50 μs, [2] = 10 μM).

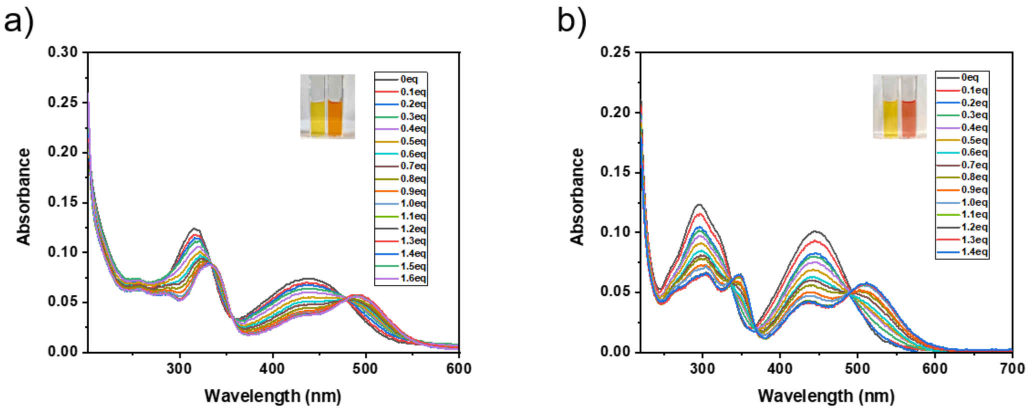

Figure S8. (a) UV–vis absorption spectra of aqueous solutions of 1 (10 μM) and CB[8] at concentrations ranging from 0 to 16 μM under ambient conditions. Inset: photographs of solutions of 1 and 1/CB[8] under ambient conditions. **(**b) UV–vis absorption spectra of aqueous solutions of 2 (10 μM) and CB[8] at concentrations ranging from 0 to 14 μM under ambient conditions. Inset: photographs of solutions of 2 and 2/CB[8] under ambient conditions.

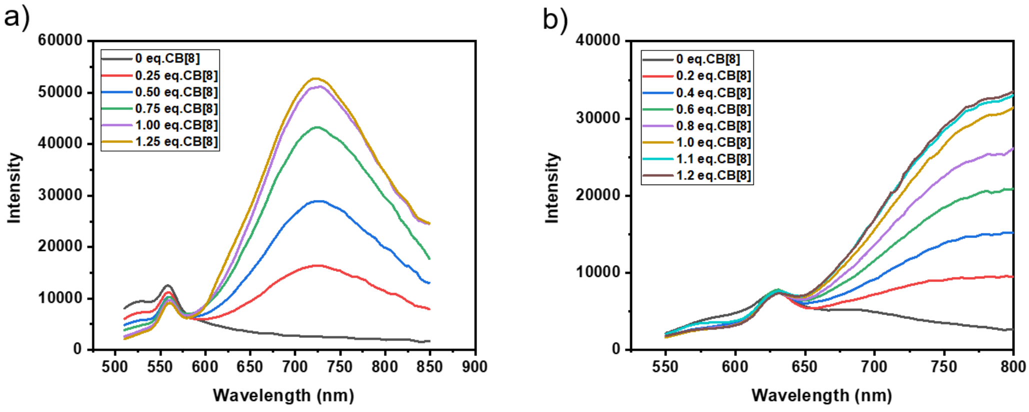

Figure S9. (a) PL spectra of aqueous solutions of 1 with different concentrations CB[8] (0 to 1.25 equiv) under ambient conditions (λ_{ex}= 495 nm, [1] = 20 μM).; (a) PL spectra of aqueous solutions of 2 with different concentrations CB[8] (0 to 1.2 equiv) under ambient conditions (λ_{ex}= 510 nm, [2] = 20 μM).

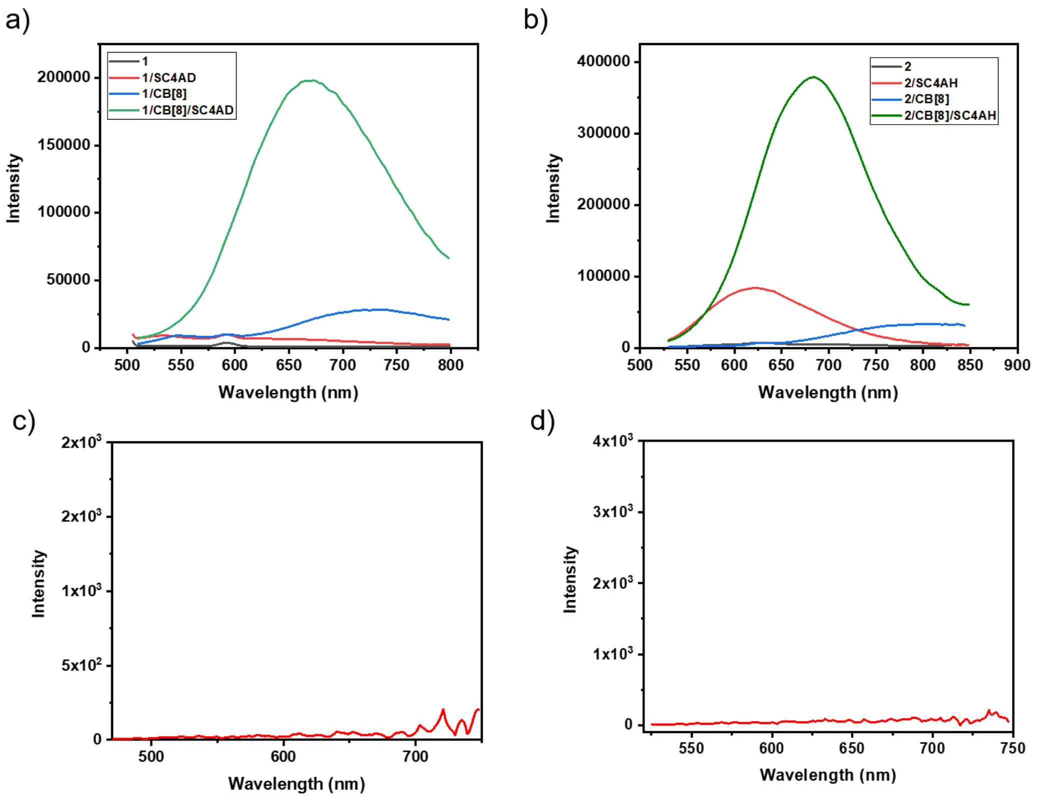

Figure S10. (a) PL spectra of aqueous solutions of 1 (20 μM, black line), 1/CB[8] ([1] = 20 μM, [CB[8]] = 20 μM, blue line), 1/SC4AD ([1] = 2 × 10^{-5} M, [SC4AD] = 20 μM, red line), 1/CB[8]/SC4AD ([1] = 20 μM, [CB[8]] = 20 μM, [SC4AD] = 20 μM, green line) under ambient conditions (λ_{ex}= 495 nm).; (b) PL spectra of aqueous solutions of 2 (20 μM, black line), 2/CB[8] ([2] = 20 μM, [CB[8]] = 20 μM, blue line), 2/SC4AD ([2] = 20 μM, [SC4AD] = 20 μM, red line), 2/CB[8]/SC4AD ([2] = 20 μM, [CB[8]] = 20 μM, [SC4AD] = 20 μM green line) under ambient conditions (λ_{ex}= 510 nm).; (c) The delayed spectrum of 1/SC4AD under ambient conditions (λ_{ex}= 495 nm, delayed time = 50 μs, [1] = 20 μM, [SC4AD] = 20 μM). (b) The delayed spectrum of 2/SC4AD under ambient conditions (λ_{ex}= 510 nm, delayed time = 50 μs, [2] = 20 μM, [SC4AD] = 20 μM).

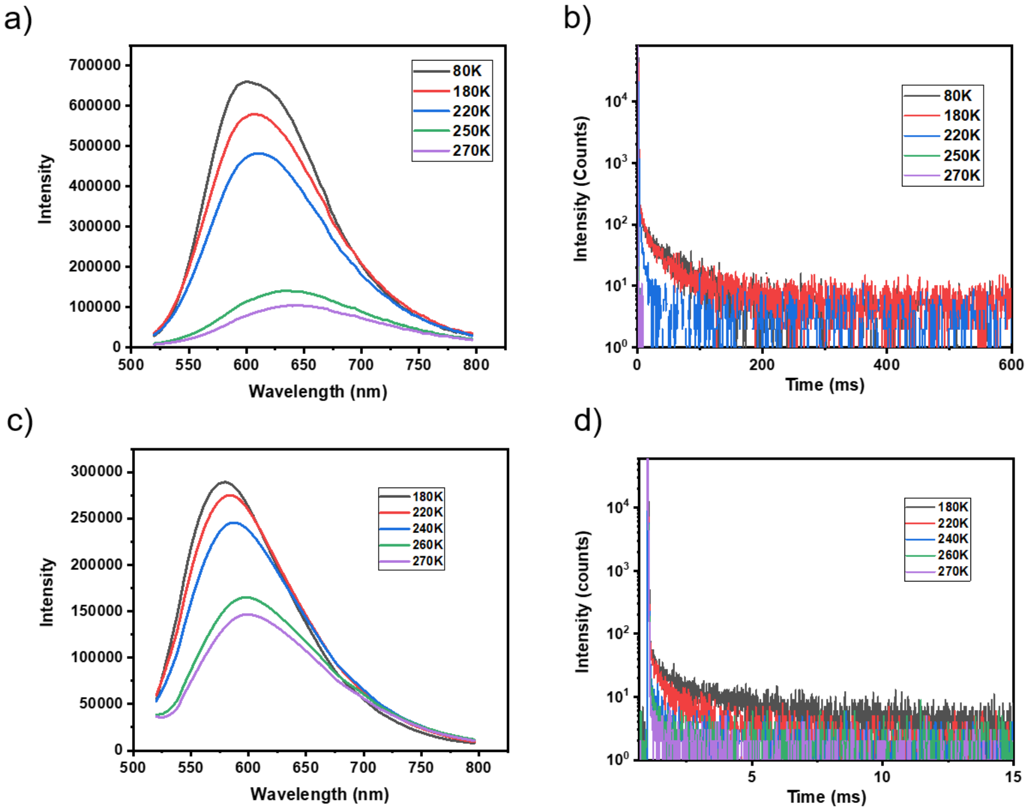

Figure S11. (a) PL spectra of 1/CB[8]/SC4AD in aqueous solution at different temperature; (b) Time-resolved photoluminescence decay spectra of 1/CB[8]/SC4AD in aqueous solution at different temperature.([1] = 20 μM, [CB[8]] = 20 μM, [SC4AD] = 20 μM, λ_{ex} = 495 nm). (c) PL spectra of 2/CB[8]/SC4AD in aqueous solution at different temperature; (d) Time-resolved photoluminescence decay spectra of 2/CB[8]/SC4AD in aqueous solution at different temperature.([2] = 20 μM, [CB[8]] = 20 μM, [SC4AD] = 20 μM, λ_{ex} = 510 nm).

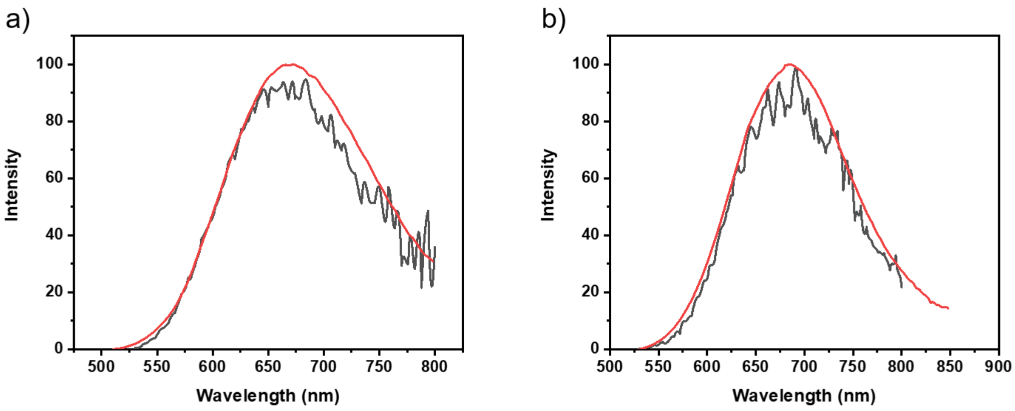

Figure S12. (a) Steady-state PL (red line) and phosphorescence spectra (black line) of 1/CB[8]/SC4AD ([1] = 20 μM, [CB[8]] = 20 μM, [SC4AD] = 20 μM, λ_{ex} = 495 nm, delayed time = 100 μs). (b) Steady-state PL (red line) and phosphorescence spectra (black line) of 2/CB[8]/SC4AD ([2] = 20 μM, [CB[8]] = 20 μM [SC4AD] = 20 μM, λ_{ex} = 510 nm, delayed time = 100 μs).

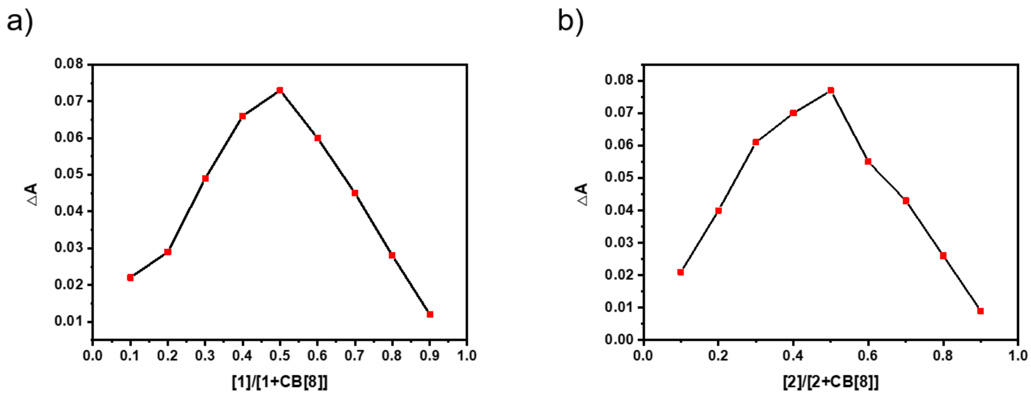

Figure S13. Job's plots show the 1:1 stoichiometry of the complex between guest and CB[8] (1 recorded the absorbance at 430 nm (a), 2 recorded the absorbance at 445 nm (b)). The total concentration is constant ([1] + [CB[8]] = 10 μM, [2] + [CB[8]] = 10 μM)

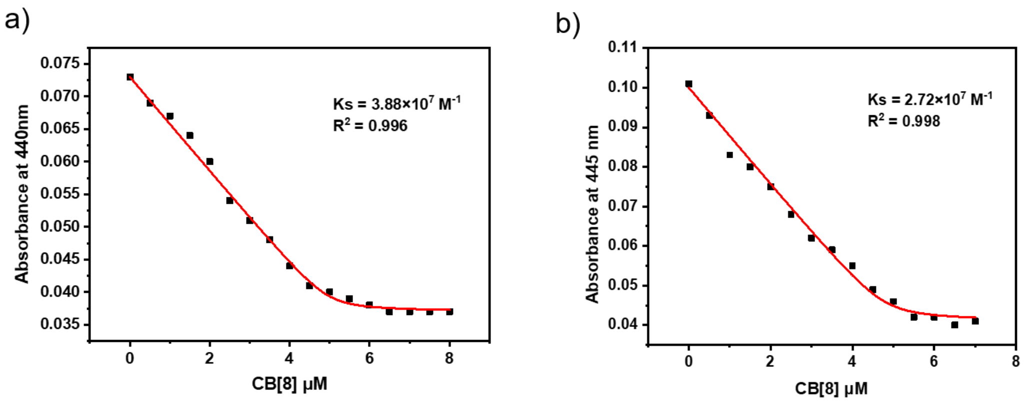

Figure S14. (a) The nonlinear least-squares analyses of the UV absorbance changes with addition 0-1.6 eq CB[8] to calculate the association constant between 1 and CB[8]. (b) The nonlinear least-squares analyses of the UV absorbance changes with addition 0-1.4 eq CB[8] to calculate the association constant between 1 and CB[8].

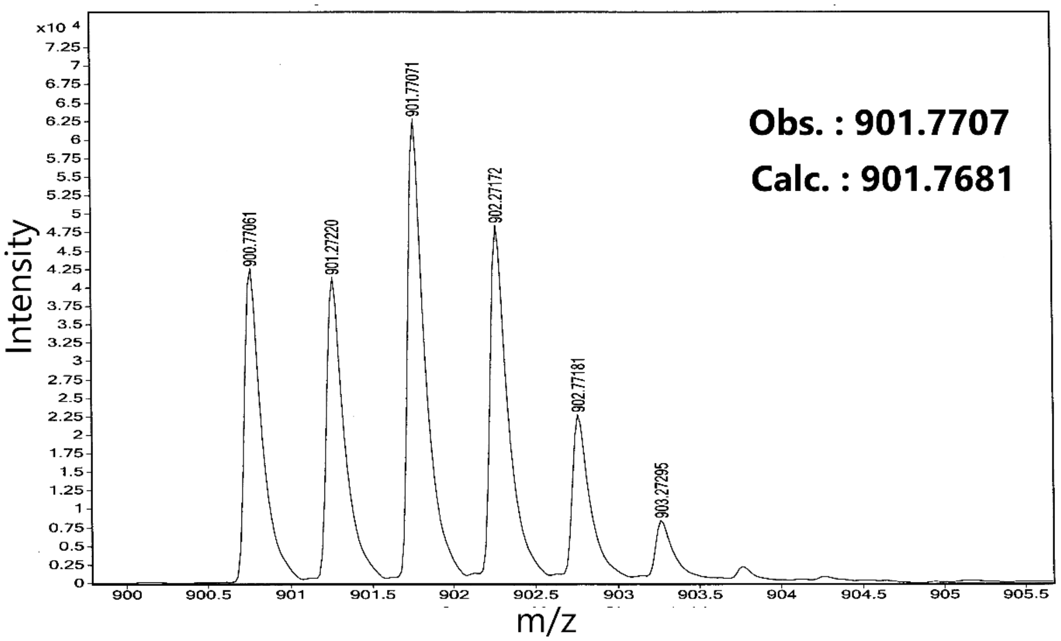

Figure S15. The high-resolution ESI-TOF MS of 1/CB[8].

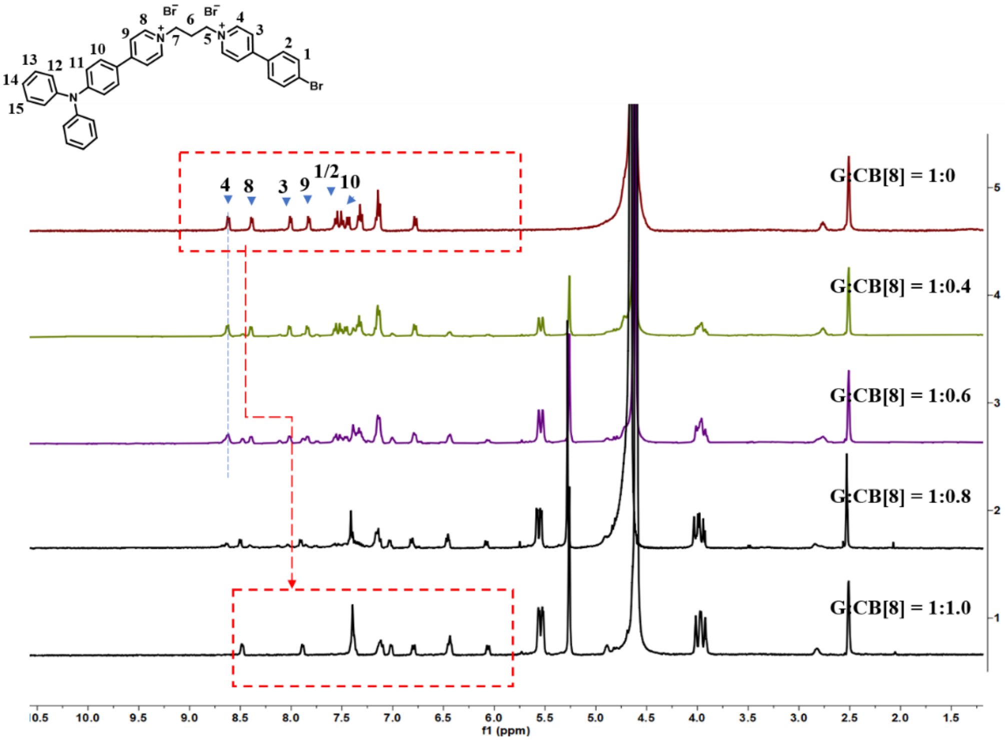

Figure S16. ^{1}H NMR spectral changes of 2 after adding 0, 0.4, 0.6, 0.8, 1.0 equiv. CB[8]. ([2] = 20 μM, 400 MHz, D_2O, 298 K).

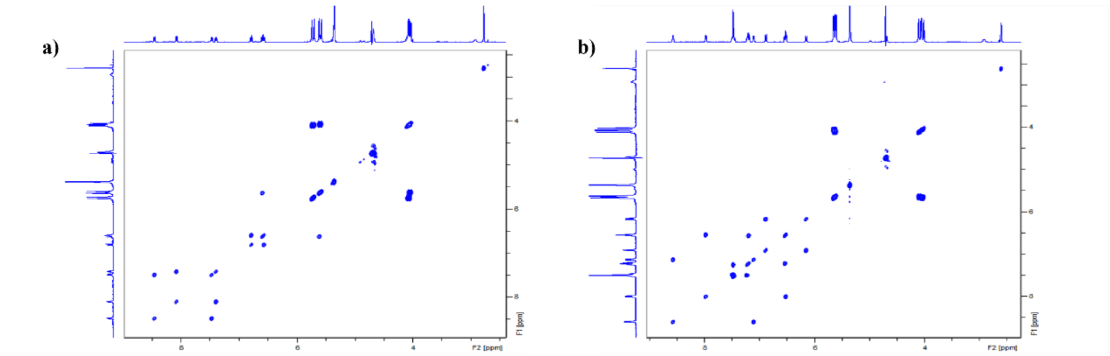

Figure S17. (a) COSY spectrum (400 MHz, D_2O, 298 K) of 1/CB[8] ([1/CB[8]] = 1 mM). (b) COSY spectrum (400 MHz, D_2O, 298 K) of 2/CB[8] ([2/CB[8]] = 1 mM).

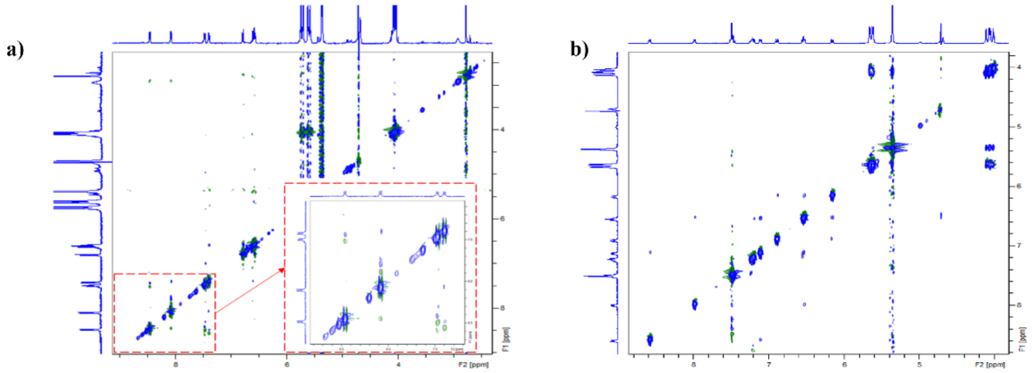

Figure S18. (a) NOESY spectrum (400 MHz, D_2O, 298 K) of 1/CB[8] ([1/CB[8]] = 1 mM). (b) NOESY spectrum (400 MHz, D_2O, 298 K) of 2/CB[8] ([2/CB[8]] = 1 mM).

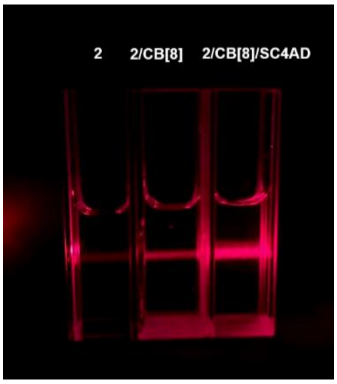

Figure S19. The Tyndall effect of 2 (left), 2/CB[8] (middle), and 2/CB[8]/SC4AD (right) ([2] = [CB[8] = [SC4AD] = 20 μM).

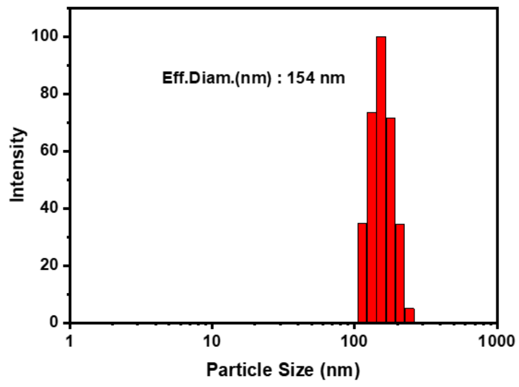

Figure S20. DLS data of 2/CB[8]/SC4AD ternary assembly ([2] = [CB[8] = [SC4AD] = 20 μM).

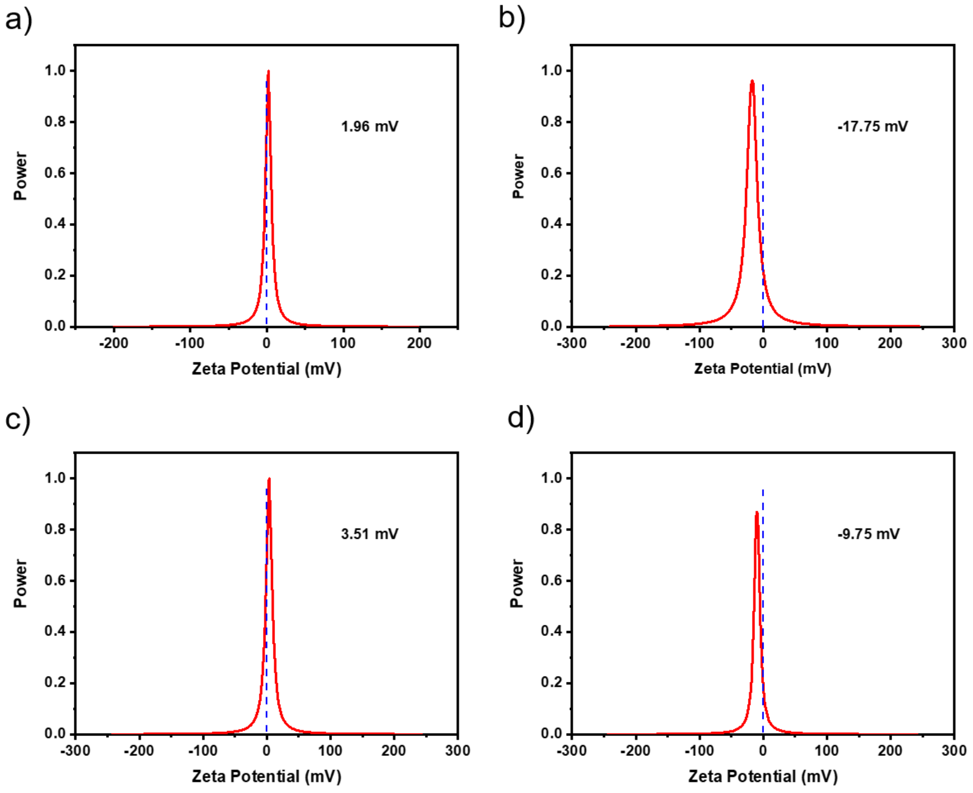

Figure S21. Zeta Potential of (a) 1/CB[8], (b) 1/CB[8]/SC4AD, (c) 2/CB[8], (d) 2/CB[8]/SC4AD ([1] = [2] = [CB[8] = [SC4AD] = 20 μM).

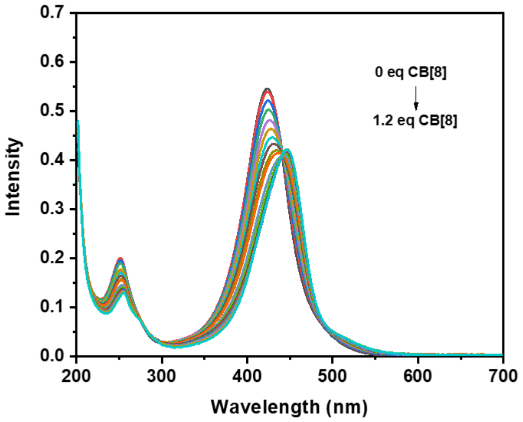

Figure S22. UV–vis absorption spectra of aqueous solutions of 3 (20 μM) and CB[8] (0 to 1.2 equiv) under ambient conditions.

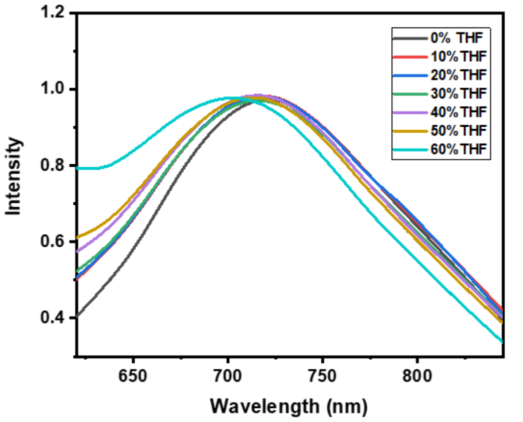

Figure S23. PL spectra of 1/CB[8] with varying concentrations of THF from 0 to 60% in aqueous solution under ambient conditions.

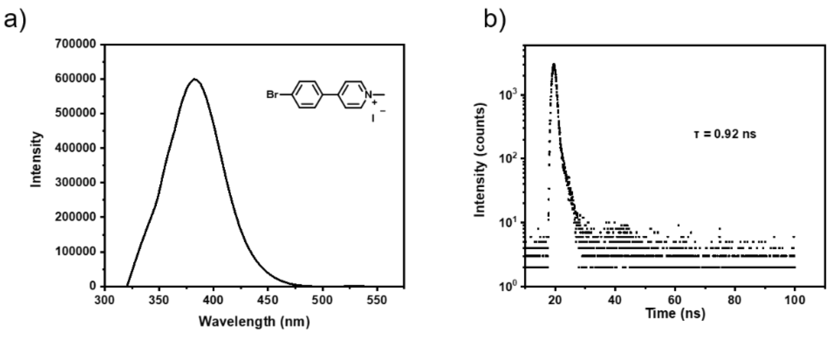

Figure S24. The steady-state spectra and time-correlated decay curves of compound 1-2 (λ_{ex}= 300 nm, [1-2] = 1 × 10^{-5} M).

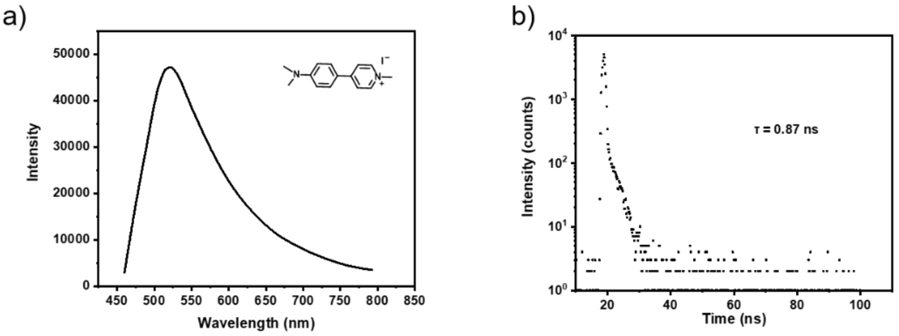

Figure S25. The steady-state spectra and time-correlated decay curves of compound 1-3 (λ_{ex}= 440 nm, [1-2] = 10 μM).

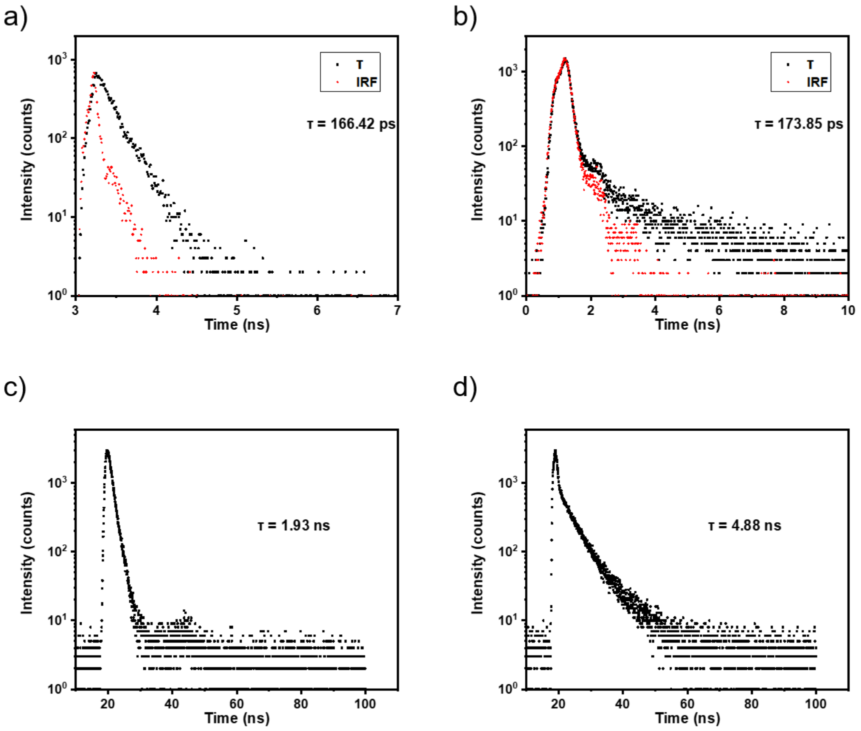

Figure S26. (a) Time-correlated decay curves of 1 in aqueous at 390 nm ([1] = 10 μM); (b) Time-correlated decay curves of 1 in aqueous at 530 nm ([1] = 10 μM); (c) Time-correlated decay curves of 1/CB[8] in aqueous at 390 nm ([1] = 10 μM); (b) Time-correlated decay curves of 1/CB[8] in aqueous at 530 nm ([1] = 10 μM).

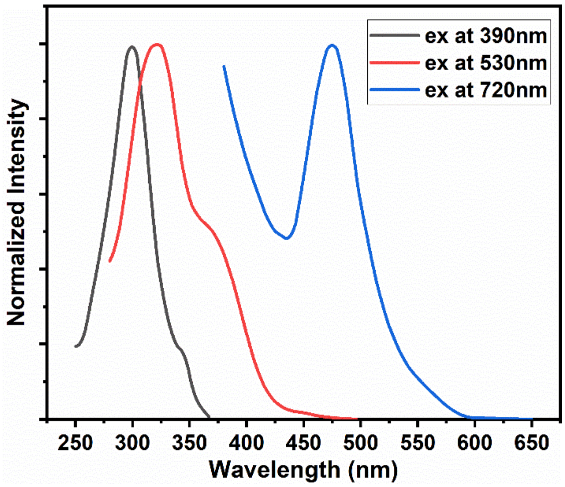

Figure S27. Excitation spectra of 1/CB[8] at 720 nm (blue), 530 nm (red), 390 nm (black).

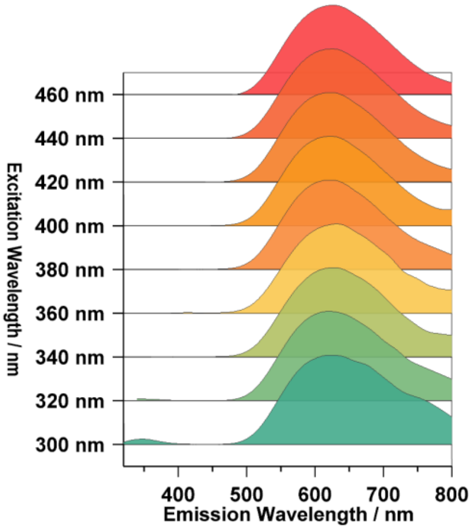

Figure S28. PL spectra of 1/CB[8]/SC4AD ([1] = [CB[8] = [SC4AD] = 20 μM) in aqueous solution under ambient conditions excited with different wavelengths (from 300 to 460 nm).

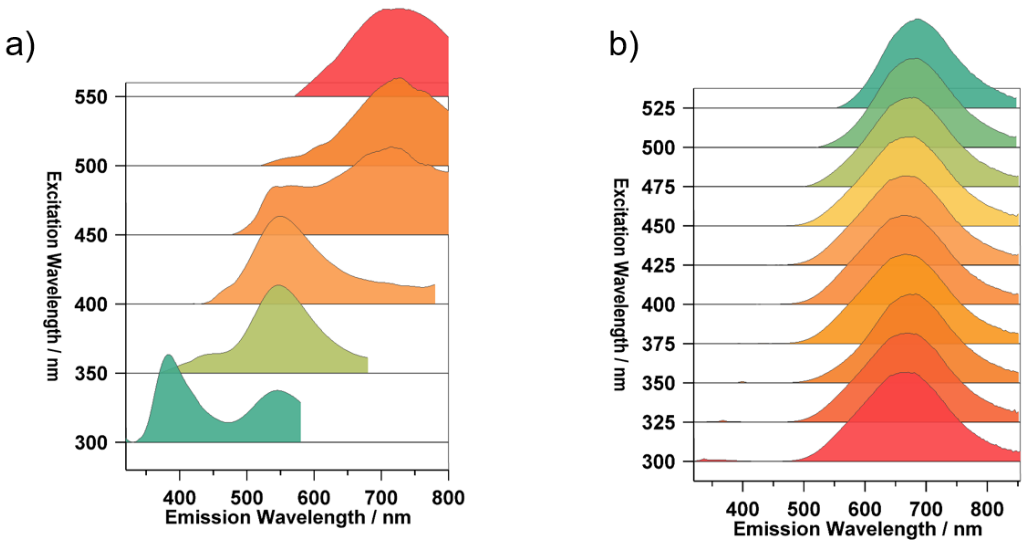

Figure S29. (a) PL spectra of 2/CB[8] ([2] = [CB[8] = 20 μM) in aqueous solution under ambient conditions excited with different wavelengths (from 300 to 550 nm). (b) PL spectra of 2/CB[8]/SC4AD ([2] = [CB[8] = [SC4AD] = 20 μM) in aqueous solution under ambient conditions excited with different wavelengths (from 300 to 525 nm).

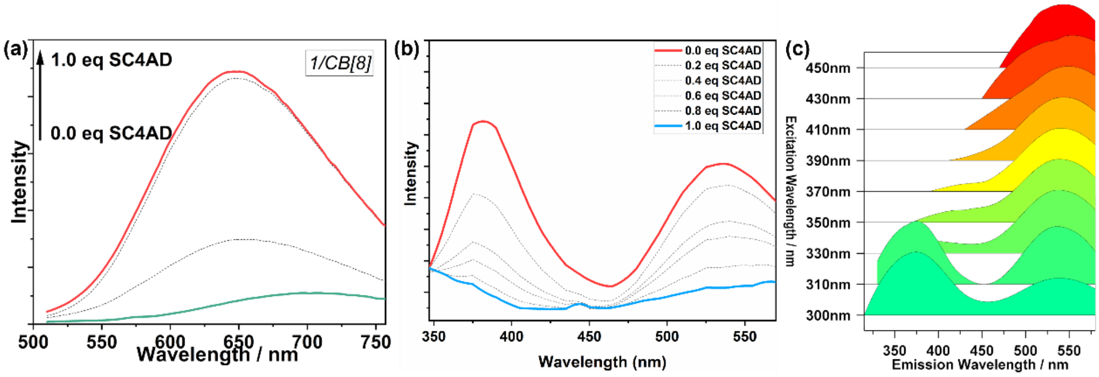

Figure S30. (a) PL spectra of 1/CB[8] upon the addition of 0, 0.5 × 10^{-5}, 1.0 × 10^{-5}, 1.2 × 10^{-5} M SC4AD in aqueous solution. (λ_{ex} of 1/CB[8] = 495 nm,). (b) PL spectra of 1/CB[8] upon the addition of 0, 0.5 × 10^{-5}, 1.0 × 10^{-5}, 1.2 × 10^{-5} M SC4AD in aqueous solution. (λ_{ex} of 1/CB[8] = 300 nm, [1] = [CB[8]] = 2 × 10^{-5} M). (c) PL spectra of 1/CB[8]/SC4AD ([1/CB[8]/SC4AD] = 2 × 10^{-5} M) in aqueous solution under ambient conditions excited with different wavelengths (from 300 to 450 nm)

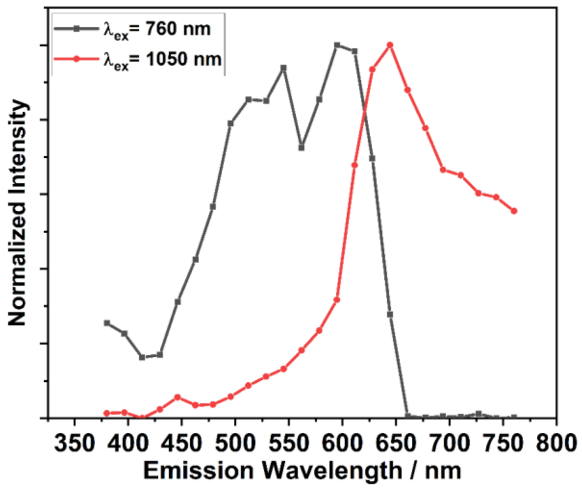

Figure S31. Two-photon excitation spectrum of 1/CB[8]/SC4AD in aqueous solution under ambition conditions ([1/CB[8]/SC4AD] = 2 × 10^{-5} M).

Section D. Theoretic Calculations

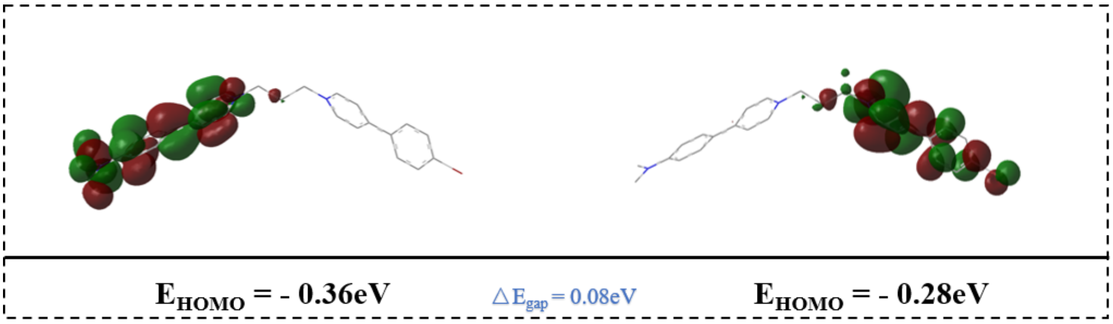

Figure S32. Optimized molecular configuration and calculated spatial distributions of HOMO and LUMO for 1.

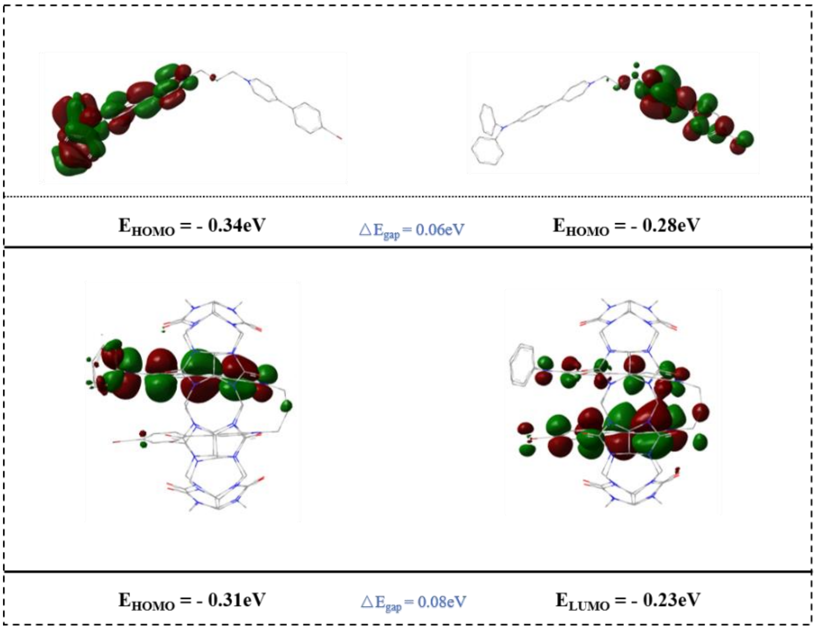

Figure S33. Optimized molecular configuration and calculated spatial distributions of HOMO and LUMO for 2 and 2/CB[8].

Section E. Cells Experiments

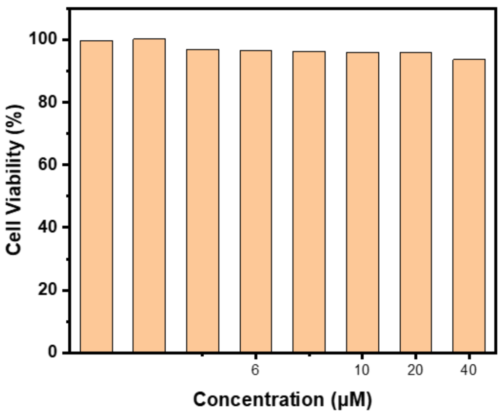

Figure S34. Cell viability of A549 cancer cells for 1/CB[8]/SC4AD at different concentrations.(0 μM, 2 μM, 4 μM, 6 μM, 8 μM, 10 μM, 20 μM, 40 μM).

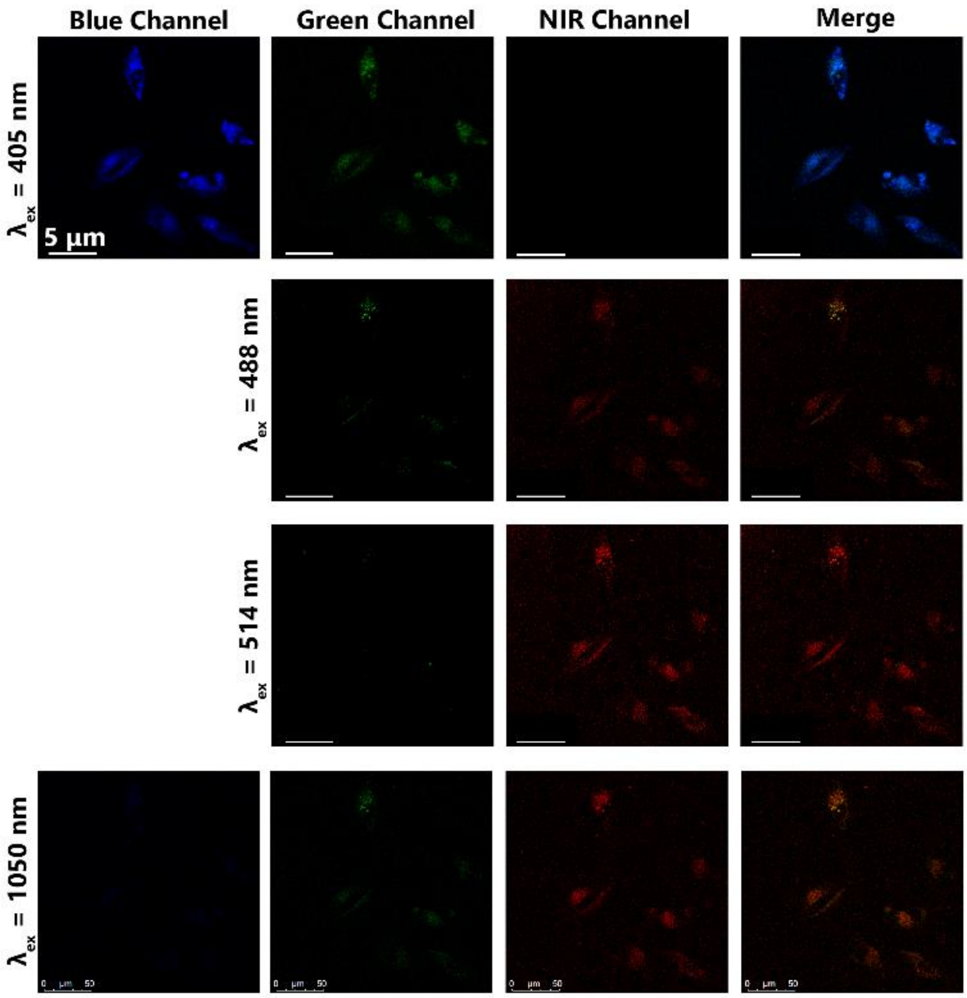

Figure S35. Cell imaging of living A549 cells incubated with 1/CB[8]/SC4AD using 405 nm, 488 nm, 514nm, and 1050 nm excitation light. Blue Channel: λ_{em} = 419-450 nm; Green Channel: λ_{em} = 450-530 nm; NIR Channel: λ_{em} = 650-750 nm.

Section F. Reference

(1) Ma, X. K.; Zhang, W.; Liu, Z. X.; Zhang, H. Y.; Zhang, B.; Liu, Y. Supramolecular Pins with Ultralong Efficient Phosphorescence. *Adv. Mater.* **2021,** *33*, 2007476.

(2) Calabrese, V.; Quici, S.; Rossi, E.; Cariati, E.; Dragonetti, C.; Roberto, D.; Tordin, E.; De Angelis, F.; Fantacci, S. Highly stable 7-N,N-dibutylamino-2-azaphenanthrene and 8-N,N-dibutylamino-2-azachrysene as a new class of second order NLO-active chromophores. *Chem. Commun.* **2010,** *4*, 8374.

(3) Wang, T.; Shah, I.; Yang, Z.; Yin, W.; Zhang, S.; Yang, Y.; Yin, P.; Ma, H. Incorporating Thiourea into Fluorescent Probes: A Reliable Strategy for Mitochondrion-Targeted

Imaging and Superoxide Anion Tracking in Living Cells. *Anal. Chem.* **2020,** *92*, 2824.

(4) Lomov, D. A.; Abramyants, M. G.; Astashkina, N. V.; Korotkikh, N. I.; Lubenets, V. I.; Novikov, V. P.; Komarovskaya-Porokhnyavets, E. Z.; Smolyar, N. N. Synthesis and Fungi/Bactericidal Activities of 4-(4-Dimethylaminophenyl)Pyridine Derivatives. *Pharm. Chem. J.* **2016,** *50*, 526.

(5) Zhang, Y.; Zhou, T.-Y.; Zhang, K.-D.; Dai, J.-L.; Zhu, Y.-Y.; Zhao, X. Encapsulation Enhanced Dimerization of a Series of 4-Aryl-N-Methylpyridinium Derivatives in Water: New Building Blocks for Self-Assembly in Aqueous Media. *Chem. Asian. J.* **2014,** *9*, 1530.
